# Q-marker identification strategies in traditional Chinese medicines: a systematic review of research from 2020 to 2024

**DOI:** 10.3389/fmed.2025.1709969

**Published:** 2026-01-16

**Authors:** Khoa Nguyen Tran, Gia Linh Mac, Yeasmin Akter Munni, In-Jun Yang

**Affiliations:** Department of Physiology, College of Korean Medicine, Dongguk University, Gyeongju, Republic of Korea

**Keywords:** BP-ANN, metabolomics, multi-dimensional network, network pharmacology, Q-marker, strategy

## Abstract

**Background:**

The concept of “quality markers” (Q-markers) has emerged as a key solution to address limitations in the evaluation and standardization of traditional herbal medicines. Despite the introduction of various Q-marker identification strategies, methodological inconsistencies and a lack of standardization continue to pose challenges.

**Objectives:**

This review aims to systematically organize and evaluate Q-marker selection strategies published over the past 5 years and propose an optimal approach based on a comparative analysis of their strengths and limitations.

**Methods:**

A comprehensive literature search was performed on the Web of Science and PubMed for studies published between January 2020 and December 2024 using keywords related to Q-marker identification in traditional prescriptions. After removing duplicates and screening for relevance, the eligible studies were systematically reviewed. Key information, including the prescription name, therapeutic targets, methodological steps for Q-marker selection, and the final identified Q-markers, was extracted and organized into summary tables. Based on the analysis, the advantages and limitations of each strategy were evaluated.

**Results:**

The studies were categorized into four representative strategies: [S1] mechanism-driven validation, which relies on network pharmacology and bioassays to align compounds with disease pathways (22 cases, 36.67%); [S2] profile–effect correlation modeling, which uses statistical and machine learning tools to link chemical composition with pharmacodynamic outcomes (24 cases, 40%); [S3] *in silico* preliminary filtering, which rapidly screens candidate compounds using computational predictions without experimental validation (8 cases, 13.33%); and [S4] multi-criteria decision frameworks, which integrate formulation hierarchy, efficacy, and chemical properties into composite scoring models (6 cases, 10.00%). The average number of Q-markers identified in each strategy was 7.23, 6.61, 8.25, and 7.5, respectively. While each strategy has unique analytical strengths, they often lack consistency and reproducibility when applied in isolation. To overcome this, we recommend a stepwise approach that integrates (1) compound selection based on bioavailability, (2) disease-relevant biomarker selection, (3) correlation modeling, and (4) a multi-criteria scoring framework based on TCM principles. This integrated model accounts for compound bioavailability, specificity, and formulation roles, enabling the identification of functionally relevant Q-markers, including low-abundance constituents.

**Conclusion:**

This review can provide valuable insights to guide future research and development of traditional herbal medicines, particularly in the context of quality control and innovative drug discovery. The proposed framework improves biological relevance and practical applicability and may serve as a scalable model for the quality assessment of multi-component herbal systems and complex pharmacological formulations.

## Graphical abstract

**Figure:**
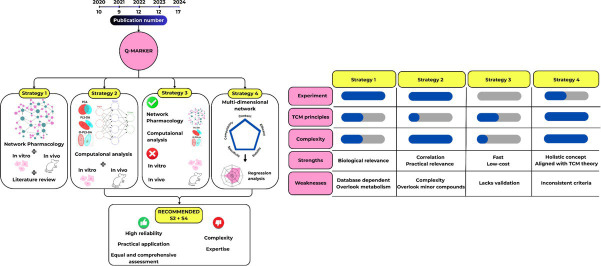

## 1 Introduction

The value of traditional herbal prescriptions (THPs) lies in their ability to provide therapeutic benefits through complex natural compounds, many of which have shown potential for integration into modern medical applications. THPs are characterized by inherent complexities, including the composition of multiple compounds and multi-target mechanisms (1). These features pose substantial challenges to achieving consistent quality control and standardization. Furthermore, the composition of THPs can vary significantly due to factors such as soil quality, climate, harvest timing, and postharvest processing techniques (2, 3).

Development strategies for THPs have frequently emphasized high-content bioactive ingredients, primarily due to practical considerations such as ease of analysis, regulatory requirements, established practices, research limitations, and economic factors. This approach often fails to correlate directly with therapeutic efficacy because it overlooks the complex interactions among the various components within a prescription that are essential to its overall effectiveness. To address these limitations, Liu et al. proposed the concept of a quality marker (Q-marker) in 2016 (4). Unlike previous approaches that focused solely on major or bioactive compounds, Q-markers comprehensively consider biological activity, absorption rate, stability, specificity, quantifiability, and compatibility based on the standard principles of traditional medicine theory (5). This innovative concept has opened new horizons in the research and application of traditional herbal medicine, leading to significant progress in quality control, efficacy evaluation, and drug development (6).

Various techniques have been developed to identify Q-markers in THPs. These approaches include computational methods (online databases, predictive tools, and mathematical models) and laboratory testing, such as absorption, stability, and bioactivity (7). The diversity of available techniques has generated multiple strategies for Q-marker determination, each with distinct advantages and limitations. While offering methodological flexibility, this multiplicity of approaches has also introduced significant ambiguities and inconsistencies in the evaluation principles. Currently, the field lacks comprehensive guidelines for selecting the most appropriate and reliable strategies for specific research contexts. Our study systematically reviews and analyzes Q-marker identification approaches published over the past 5 years, thereby suggesting suitable strategies for future studies and contributing to the quality control and standardization of THPs.

## 2 Materials and methods

This systematic review was conducted according to the updated guidelines of the Preferred Reporting Items for Systematic Review and Meta-Analyses (PRISMA) 2020 (8). The Web of Science and PubMed databases were accessed (until January 01, 2025) to search for relevant publications from 2020 to 2024, using the search term: “*[(q-marker) OR (quality marker)] AND [(herbal) OR (traditional Chinese medicine) OR (extract) OR (prescription)] NOT (review)*.”

First, duplicate articles were removed using Microsoft Excel. In the first round of screening, the researchers checked the title and abstract of each article to eliminate records that did not meet the following criteria (1): using traditional prescriptions (2), studying quality markers related to traditional herbal medicines, and (3) specifically providing strategies for selecting quality markers. In the second round, the full text of the remaining articles was evaluated using the following exclusion criteria (1): not specifically mentioning the names of quality markers (2), single-step strategy, and (3) full text not accessible. A single-step strategy indicates studies using only one approach for Q-marker identification, such as only HPLC fingerprinting or only network pharmacology. Studies that combine network pharmacology and molecular docking were classified as “network-pharmacology-only” if they did not include any wet-lab experiments (bioactivity, serum absorption, chromatographic validation). All included studies must employ a multi-step strategy that integrates more than two different analytical procedures, such as computational screening and experimental validation or chromatographic analysis, statistical modeling, and bioactivity testing. Upon confirmation of the final records, the information from each record was extracted into tables, including prescription name, targets, step-by-step strategy for selecting quality markers, and specific final quality markers. All steps in this process were performed by two independent researchers, while a third researcher was consulted in case of disagreement.

## 3 Results

**Figure 1:**
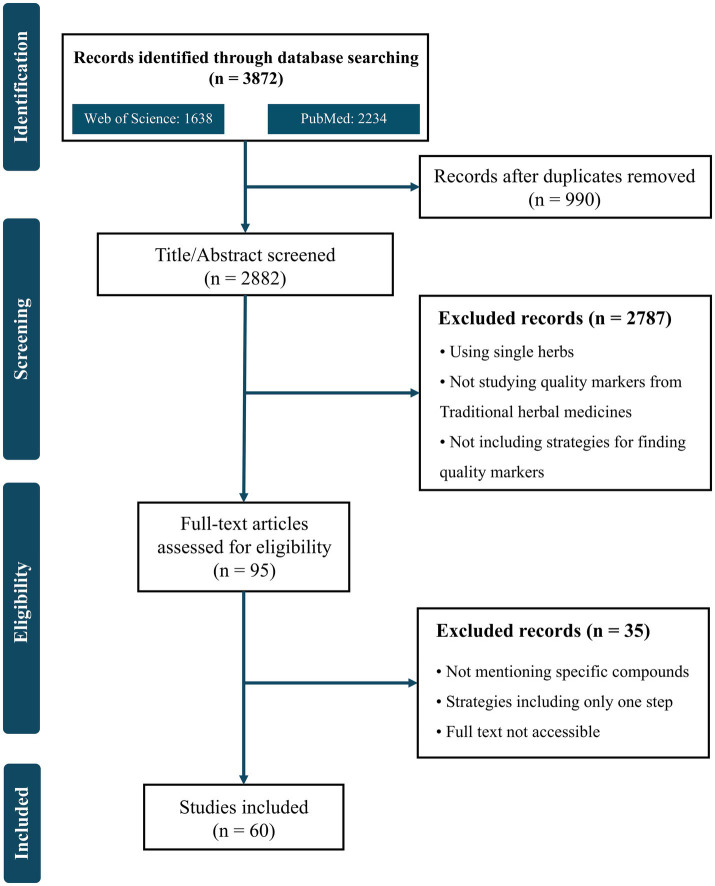
Flow diagram for the study selection in this systematic review.

Using specific search terms, we collected 1,638 records from the Web of Science and 2,234 records from PubMed from January 2020 to December 2024, from which 990 duplicates were eliminated. After screening 2,882 abstracts, we excluded 2,787 abstracts that did not meet our eligibility criteria, including (1) using a prescription (2), studying Q-markers in traditional Chinese medicine (TCM) areas, and (3) specifying a strategy for Q-marker identification. In the full-text evaluation round, we further removed 35 studies belonging to the following categories (1) (1): nonspecific compounds mentioned (2), single-step strategy used, and (3) full text not accessible. Finally, 60 studies were selected for this review (Figure 1).

**Figure 2:**
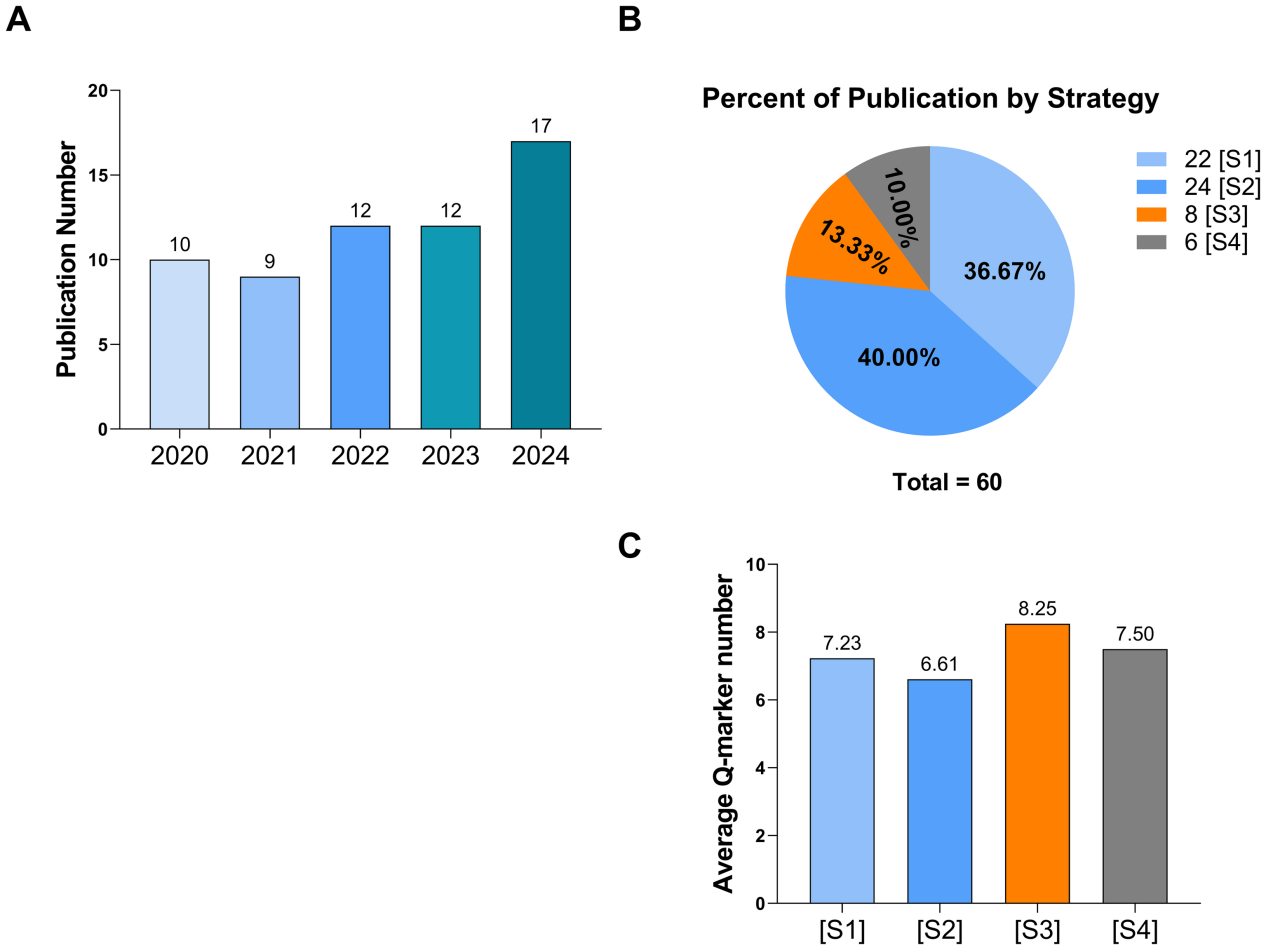
Number of publications regarding **(A)** Q-marker identification for THPs from 2020 to 2024, together with **(B)** their classification into different strategies, and **(C)** the corresponding average Q-marker number as outcomes.

Of the 60 selected studies, 19 were published in the first 2 years, followed by 24 studies from 2022 to 2023. The highest number of publications (9) was recorded in 2024 (Figure 2A). As shown in Table 1, these studies, based on their core methodological characteristics, were classified into four strategies: [S1] Mechanism-driven validation strategy (22 cases, accounting for 36.67%); [S2] Profile–Effect correlation strategy (24 cases, 40.00%); [S3] *In silico* preliminary filtering strategy (8 cases, 13.33%); and [S4] Multi-criteria decision framework strategy (6 cases, 10.00%) (Figure 2B, Table 1). Although some studies involved overlapping features between strategies, the classification was based on the predominant methodology used. Across the four strategies, the average number of Q-markers identified varied. [S1] reported an average of 7.23 Q-markers per study, while [S2] yielded an average of 6.61. [S3] showed the highest average, at 8.25 Q-markers, and [S4] reported an average of 7.50 Q-markers (Figure 2C).

**Table 1:** Classification criteria for the four research strategies.

Strategy	Classification criteria
Strategy 1 [S1]	Relied on building disease–compound–target networks and validating those connections through experiments to understand the biological mechanisms involved
Strategy 2 [S2]	Relied on analyzing the relationship between the material’s compositions and its effects, predominantly applying computational/statistical models (e.g., spectrum–bioactivity relationship (SBR), plotting of correlation between marker metabolites and serum components (PCMS), back-propagation artificial neural network (BP-ANN)) with laboratory experiments
Strategy 3 [S3]	Relied solely on *in silico* analysis (e.g., database mining and molecular docking) without any experimental validation
Strategy 4 [S4]	Applied a multi-dimensional evaluation framework based on the “five principles” of TCM, integrating all dimensions through scoring systems, such as regression area (RA) or shaded area (QMI)

### 3.1 Strategy 1: mechanism-driven validation strategy (22)

**Figure 3:**
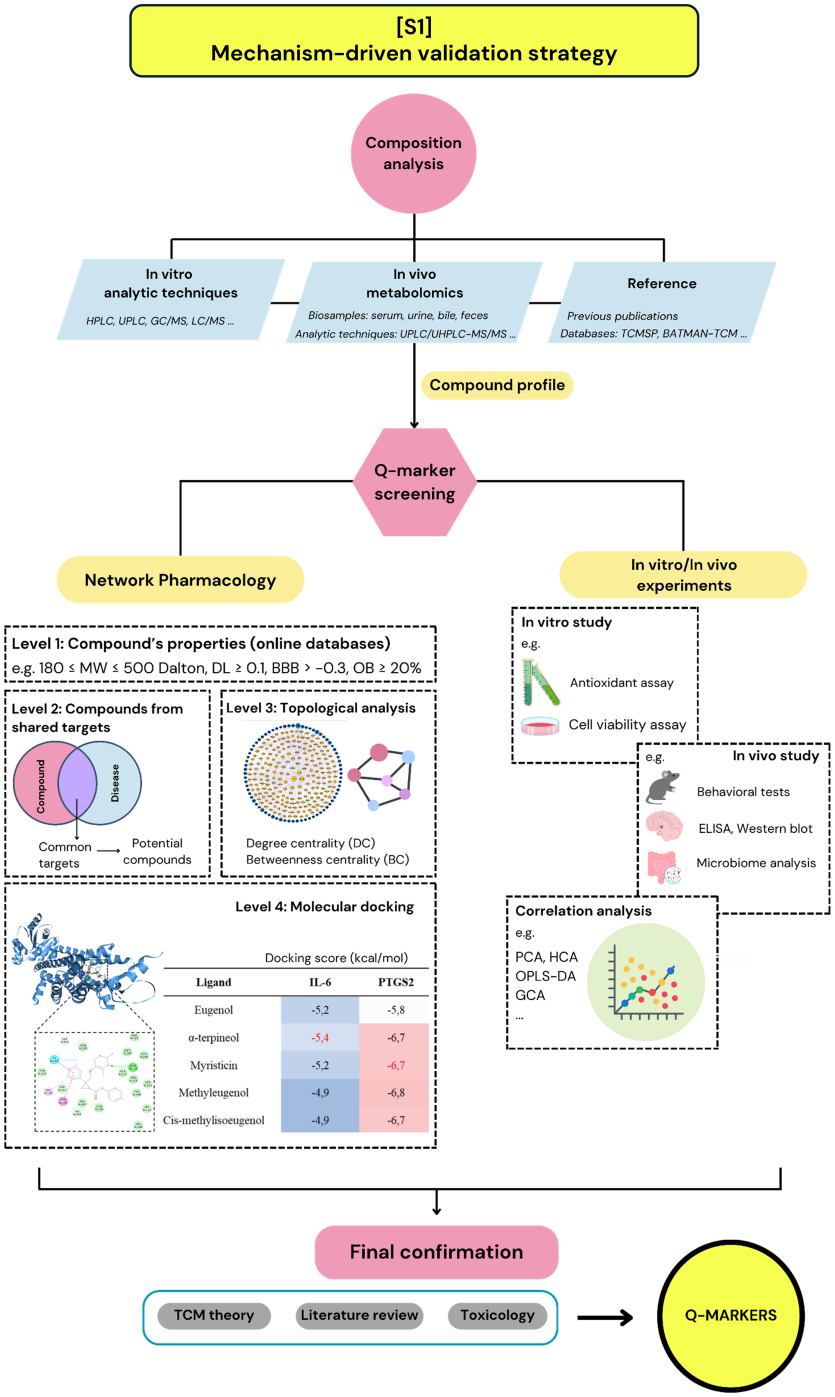
The demonstration of strategy 1: mechanism-driven validation strategy.

A total of 22 studies were classified under this group. Overall, this strategy integrates a wide range of methods to identify Q-markers, including extract composition analysis, metabolomics, network pharmacology, molecular docking, and cell- or animal-based validation experiments. Network pharmacology was employed in all studies, although it was utilized at different stages and for varying purposes depending on the specific research design (Figure 3).

#### 3.1.1 Composition analysis

Identifying the compound profiles of herbal extracts was the initial step in any Q-marker determination strategy; however, this step can be performed using different methods. According to the 22 studies, extract composition was obtained through three main approaches (1): direct analysis of the extraction fluid using techniques such as high-performance liquid chromatography (HPLC), ultra-performance liquid chromatography (UPLC), gas chromatography–mass spectrometry (GC–MS), and liquid chromatography–mass spectrometry (LC–MS) (2); indirect identification based on *in vivo* absorption analysis using biological samples such as urine, plasma, or serum; and (3) referencing compound data from online databases or previously published literature.

HPLC, UPLC, GC–MS, and LC–MS are well-established analytical techniques, each offering distinct advantages in separating, identifying, and characterizing complex chemical mixtures based on their physicochemical properties (10). As shown in Table 1, these techniques are rarely applied in isolation; rather, they are often integrated with advanced detectors or mass spectrometers to enhance sensitivity, resolution, and structural elucidation capabilities. Common combinations include HPLC with diode array detection (HPLC-DAD), ultra-high-performance liquid chromatography (UPLC), or HPLC with quadrupole time-of-flight mass spectrometry (UPLC/HPLC-QToF-MS/MS), and ultra-high performance liquid chromatography coupled with Q-Exactive Orbitrap mass spectrometry (UHPLC-Q-Exactive-Orbitrap-MS). The resulting chromatographic or spectrometric fingerprints served as chemical profiles, reflecting both the qualitative and semi-quantitative compositions of the extract. Each peak in the chromatogram corresponded to a specific compound, enabling comprehensive mapping of the phytochemical profile of the extract.

Metabolomic analysis, frequently encountered in our dataset, was used for profiling small-molecule metabolites in biological samples from animal subjects following a defined period of drug administration. Metabolites extracted from biosamples such as plasma, serum, urine, bile, and feces were commonly analyzed using advanced techniques like UPLC-MS/MS or UHPLC–MS/MS. The resulting complex spectral data were compared against spectral libraries or databases to identify and quantify the target metabolites. Based on the principle that compounds must be absorbed to exert their action, metabolomics was used not only for profiling drug metabolism but also as a screening tool to eliminate compounds with low bioavailability or negligible systemic exposure. For instance, in study A17, an initial chemical analysis of Guchang Zhixie pills identified 198 compounds. However, following metabolomic profiling, only 17 well-absorbed compounds detected in plasma were selected as candidate Q-markers for further investigation.

The final method for primary component analysis involved gathering data from online databases, such as the Traditional Chinese Medicine Systems Pharmacology Database and Analysis Platform (TCMSP), the Traditional Chinese Medicine Integrated Database (TCMID), Herb Ingredients’ Targets (HIT), and the Bioinformatics Analysis Tool for Molecular Mechanism of TCM (BATMAN-TCM), which integrate vast amounts of information on herbs and their ingredients. Four of the 22 studies in [S1] utilized this approach to acquire the compound profiles of the herbs. For example, the Lianhua Qingwen capsule (study A06) contained 13 medicinal herbs, and data collected from TCMSP and TDT provided a total of 538 compounds. Similarly, study A02 on Da-Cheng-Qi Decoction (DCQD), which includes four crude herbs, identified 272 potential constituents in three databases: TCMID, TCMSP, and HIT.

#### 3.1.2 Network pharmacology

Network pharmacology was widely used in the reviewed studies to explore interactions between compounds and biological networks. Researchers utilized various online databases (Table 2) to identify compounds, predict their molecular targets, and map associated biological pathways (11). The resulting networks enabled visualization of compound–target–pathway interactions and supported the prioritization of potentially bioactive compounds. In these studies, network pharmacology analyses were typically applied at four hierarchical levels.

**Table 2:** Strategy 1: mechanism-driven validation strategy.

Code	Publication year	Materials	Target	Process					Q-marker results	Reference
				Step 1	Step 2	Step 3	Step 4	Step 5		
A01	2020	Guan-Xin-Jing Capsule	Cardiovascular diseases	UHPLC-QTOF-MS/MS	Selection: intensity > 10,000, OB > 30%, DL > 0.18	Compound target: Swiss Disease targets: OMIM, TTD, GAD, PharmGkb	Selection based on pharmacopoeia quantitative indicators, pharmacokinetic parameters, and target similarities	UHPLC-QTOF-MS/MS + PCA + PLS-DA	3-n-butylphthalide, salvianolic acid G, ginsenoside Rg1, albiflorin, cryptotanshinone, paeoniflorin, tanshinone IIA, ligustlide, tanshinone IIB, tokinolide B, and salvianolic acid H	(57)
A02	2020	Da-Cheng-Qi Decoction	Intestinal obstruction	Network pharmacology: Compounds + Compound targets: TCMID, TCMSP, HIT Disease targets: GAD, DisGeNET, STITCH	Measurable assay: LC–MS, GC–MS, HPLC-SPD Importance assay: node degree > mean value in C-T network Effectiveness assay: literature review	Validation *In vitro*: intestine damage repair, intestinal motility, and inflammation *In vivo*: Vincristine-induced paralytic ileus rat model			Emodin, physcion, aloe-emodin, rhein, chrysophanol, gallic acid, magnolol, honokiol, naringenin, tangeretin, and nobiletin	(13)
A03	2020	Wu Ji Bai Feng Pill	Primary dysmenorrhea	Bi-directional transport studies + UPLC–MS/MS	Network pharmacology Compound targets: TCMSP, SwissTargetPrediction Disease targets: Therapeutic Target Database, DrugBank database, NCBI Gene Database, DisGeNet, GeneCards	Molecular docking			Formononetin, ferulic acid, isoliquiritigenin, neo cryptotanshinone, and senkyunolide A	(15)
A04	2020	Qingzao Jiufei	Acute lung injury	UHPLC-ESI-Q/TOF-MS	Network pharmacology Target: SwissTargetPrediction, SEA, TTD, DrugBank	Plasma metabolomics + PCA	Five determination principles		Chlorogenic acid, methylophiopogonanone A, methylophiopogonanone B, sesamin, ursolic acid, amygdalin, liquiritin apioside, liquiritigenin, and iso liquiritin	(22)
A05	2021	Chaiqin Chengqi Decoction	Acute pancreatitis	UHPLC-Q Orbitrap/MS + Principal component analysis + Hierarchical clustering analysis	Plasma metabolomics	Network pharmacology Compound targets: ETCM, STITCH, Swiss Disease targets: NCBI, DisGeNet, HPO, OMIM	*In vitro* study (pancreatic acinar cell necrosis) + Pearson correlation analysis		Emodin, rhein, aloe emodin, magnolol, hesperidin, synephrine, baicalein, and geniposide	(58)
A06	2021	Lianhua Qingwen Capsule	Influenza	Network pharmacology Compounds: TCMSP, TCM Database@Taiwan Compound targets: TCMSP, Pharmmapper Database Disease target: OMIM	UPLC	Correlation Analysis: Spectrum-Effect	Validation: anti-inflammatory activity		Chlorogenic acid, isochlorogenic acid B, and isochlorogenic acid C	(43)
A07	2022	Xinkeshu Tablets	Cardiovascular diseases	HPLC–MS/MS	*In vivo* (zebrafish model) + OSC–PLS	Network pharmacology Compound targets: Swiss, PubChem, STITCH Disease targets: OMIM, DrugBank, PharmGKB, KEGG databases	Plasma metabolomics + UHPLC/MS–MS	Five determination principles	Danshensu, salvianolic acid A, salvianolic acid B, daidzein, and puerarin	(12)
A08	2022	The Shuangshen Pingfei Formula	Idiopathic pulmonary fibrosis	UHPLC-ESI-QTOF-MS/MS	Network pharmacology Compound targets: DrugBank Disease targets: Therapeutic Target Database and literature	*In vitro*: macrophages *In vivo*: pulmonary fibrotic rat model	Plasma metabolomics + UPLC-MS/MS	Confirmation: theory of ‘Jun-chen-zuo-shi’ + quality transfer and traceability	Mangiferin, salvianolic acid B, tanshinone IIA, naringin, and glycyrrhizic acid	(21)
A09	2022	A Herbal Pair	Cardiovascular disease	Compounds: TCMSP, TCMID, Shanghai Institute of Organic Chemistry of CAS Chemistry Database Compound targets: TCMSP, PubChem, and SEA	Chemical cluster (CC) Network + Functional module (FM) network + BMCT network	Network analysis: “Two-step” Algorithm (CC-FM linkage)	*In vitro*: Thrombin inhibitory activity assay	Content validation: HPLC	Tanshinone I, tanshinone IIA, cryptotanshinone, salvianolic acid B, ferulic acid, salvianolic acid A, rosmarinic acid, chlorogenic acid, and coniferyl ferulate	(42)
A10	2022	Tongsaimai Tablet	Atherosclerosis	Serum metabolomics + UPLC-Q-Exactive Orbitrap/MS	Network Pharmacology Compound targets: ChemSpider, PubChem, SwissTargetPrediction Disease target: GeneCards	Degree of network, professional knowledge, literature validation			Ferulic acid, liquiritin, senkyunolide I, luteolin and glycyrrhizic acid	(59)
A11	2022	Kaihoujian Spray	Inflammation	HPLC/Q-TOF-MS/MS	*In vitro*: RAW264.7 Cells	Gray correlation analysis: HPLC-DAD	Network pharmacology Compound targets: SwissTargetPrediction, TCMSP, Dragbank and Uniprot Disease target: DigSee		Bergenin, sophocarpidine, sophocarpine, and trifolirhizin	(60)
A12	2023	Danlou Tablet	Coronary heart disease	Metabolomics (biosamples) + UHPLC-Q-TOF/MS, UHPLC-TQ-MS	Network pharmacology PK marker: NCBI PubChem, SwissTargetPrediction Diseases target: DrugBank, OMIM, and GeneCards	*In vitro*: H9c2 cardiomyocyte			Puerarin, alisol A, daidzein, paeoniflorin, and tanshinone IIA	(9)
A13	2023	Simiao Yong’an Decoction	Sepsis	Serum metabolomics + Linear-Trap-LC/MS^n	Network pharmacology Compound targets: TCMSP, SwissTargetPrediction, and literature and Chinese Pharmacopoeia Disease targets: GeneCards and OMIM	Molecular docking			Sweroside, chlorogenic acid, angoroside C, harpagide, ferulic acid, and glycyrrhizic acid	(19)
A14	2023	Xiaoer Chaige Tuire Oral Liquid	Influenza	*In vitro*: metabolic models, Caco-2 cell + HPLC-DAD	Serum metabolomics + UPLC-QExactive-HF-x-Orbitrap-MS	*In vitro*: Anti-inflammatory test	Network pharmacology Compound targets: TCMSP Disease targets: GeneCards and OMIM	Molecular docking	Puerarin, daidzein, benzoic acid, baicalin, baicalein, wogonoside, wogonin, oroxylin A, 3′-methoxypuerarin, paeoniflorin, scopoletin, and liquiritigenin	(61)
A15	2023	Dachaihu Decoction	Inflammation	HPLC	Network Pharmacology Compound targets: TCMSP Disease targets: GeneCards, ComparativeToxicogenomics Database, DisGeNET	Molecular docking	Effectiveness assay: RT-qPCR: inflammatory genes *In vitro*: RAW264.7 cells *In vivo*: cholecystitis model		Naringin, hesperidin, neohesperidin, baicalin, wogonoside, baicalein, and saikosaponin B2	(20)
A16	2023	Suanzaoren Decoction	Chronic restraint stress	UHPLC-Q-TOF-MS	Serum metabolomics + UHPLC-Q-TOF-MS + PCA + OPLS-DA	Network pharmacology Compound targets: TCMIP, TCMSP, and SwissTargetPrediction Disease targets: TTD, DisGeNET, GeneCards, and OMIM	Molecular docking		Coclaurine, magnoflorine, spinosin, JuA, JuB, betulinic acid, timosaponin BIII, Z-ligustilide, liquiritin, glycyrrhizic acid, and liquiritigenin	(62)
A17	2024	Guchang Zhixie Pills	Irritable bowel syndrome	UHPLC-Q-Exactive-Orbitrap-MS	Plasma metabolomics	Network pharmacology: Compound targets: PubChem, TCMSP, and SwissTargetPrediction Disease targets: PharmGKB database, GeneCards, DisGeNET, and TTD	Fingerprint analysis: HPLC, HCA, PCA, and OPLS-DA		5-HMF, magnoflorine, chlorogenic acid, tetrahydropalmatine, narcotine hydrochloride, corydaline, and berberine hydrochloride	(63)
A18	2024	Qishen Yiqi Dripping pills	Myocardial ischemia	UHPLC-Q Orbitrap HRMS	Serum metabolomics + GC/MS	Network pharmacology Compound targets: SwissTargetPrediction, PharmMapper, and TCMSP Disease targets: DrugBank, OMIM, DisGeNET, and GeneCards	Topological analysis		Astragaloside IV, ononin, calycosin, formononetin, rosmarinic acid, cryptotanshinone, salvianolic acid A, tanshinol, ginseno side Rb1, ginsenoside Rg1, nerolidol, and santalol	(64)
A19	2024	Pitongshu	Functional dyspepsia	Serum metabolomics + LC-QTOF-MS	Network Pharmacology Compound targets: TCMSP, SwissTargetPrediction Disease targets: GeneCards, OMIM	Topological analysis	Molecular docking		Hesperidin, neohesperidin, naringin, paeoniflorin, magnolol, and honokiol	(65)
A20	2024	Mailuoshutong Pill	Thromboangiitis obliterans	Serum metabolomics + UHPLC-Q-Orbitrap HRMS	Network Pharmacology Compound targets: TCMSP, SwissTargetPrediction, PharmMapper Disease targets: OMIM, TTD, GeneCards, DisGeNET	UPLC			Chlorogenic acid, paeoniflorin, liquiritin, calycosin-7-glucoside, berberine, and formononetin	(66)
A21	2024	Zhishi-Xiebai-Guizhi Decoction	Coronary heart disease	UHPLC-Q/TOF-MS UHPLC-TQ-MS	Metabolomics (biosamples) + UHPLC-Q/TOF-MS	Network Pharmacology Compound targets: SwissTargetPrediction Disease targets: DrugBank, GeneCards, OMIM	Topological analysis	*In vitro*: myocardial protection activities	Honokiol, magnolol, naringenin, magnoflorine, hesperidin, hesperetin, naringin, neohesperidin, and narirutin	(67)
A22	2024	Jiuwei Jiangtang Oral Liquid	Type 2 diabetes mellitus	Network Pharmacology Herbal components: TCMSP, ETCM, and HERB Compound target: SwissTargetPrediction Disease target: GeneCards	Topological analysis	Fingerprint analysis: HPLC, CA, PCA, and PLS-DA	*In vitro* glucose consumption and α-glucosidase inhibitory activity		Puerarin, ellagic acid, and calycosin	(68)

First, the TCMSP, TCMID, and BATMAN-TCM databases were used to filter compounds based on pharmacokinetic parameters, including molecular weight (MW), drug-likeness (DL), blood–brain barrier (BBB) permeability, and oral bioavailability (OB). For example, TCMSP suggests screening thresholds of 180 ≤ MW ≤ 500 Da, DL ≥ 0.1, BBB > −0.3, and OB ≥ 20%. These parameters are often customized depending on the experimental conditions. In study A01, UHPLC-QTOF-MS/MS analysis identified 148 compounds in the Guan-Xin-Jing extract, of which 46 compounds met the adjusted screening criteria (OB > 30%, DL > 0.18) and were retained for further analysis (Zhang et al., 2020a).

Second, network pharmacology was commonly used to identify overlapping targets between herbal compounds and disease-related genes. For example, in study A07, 62 compounds from Xinkeshu tablets (XKST) were linked to 519 predicted targets using SwissTargetPrediction, PubChem, and STITCH. Disease-associated genes for cardiovascular conditions (n = 275) were retrieved from the OMIM, DrugBank, PharmGKB, and KEGG databases. Intersecting these datasets yielded 62 common targets, and 44 compounds were selected based on this overlap (12). A similar approach was taken in study A02, whereby 82 shared targets between the extract’s compounds and the disease were identified, along with 182 corresponding compounds, followed by chemical analysis (LC/MS, GC/MS, and HPLC/SPD), which confirmed the presence of only 25 compounds in the decoction (13).

At the third level, screening was performed through a topological analysis of the compound–target network to identify key nodes and interactions. This approach evaluated network metrics, such as degree centrality (DC), betweenness centrality (BC), and closeness centrality (CC), to identify influential compounds. Degree centrality refers to the number of direct connections that a node has with other nodes. Nodes with a high degree of centrality are considered hubs because of their numerous direct interactions, indicating their potential importance in the network’s immediate connectivity and influence (14). BC measures the extent to which a node lies on the shortest paths between other nodes, acting as a critical connector or bridge within the network, while CC measures how quickly a node can reach all other nodes. Compounds with high centrality values are more likely to modulate disease-relevant biological pathways (14). In the selected cases, Cytoscape software was used for topological analysis, with DC always considered, while the use of other metrics varied. There was no standardized threshold for centrality values, which were typically determined relative to a specific network structure. For instance, studies A02, A03, A12, and A15 selected compounds that exceeded the median values of DC or BC. In contrast, studies C05 and C01 prioritized compounds with the top six and eight DC scores, respectively (9, 13, 15–18).

The final level involved molecular docking, a computational technique used to predict the binding affinity and interaction sites between compounds and target proteins. This method evaluates how strongly a compound (ligand) can bind to a protein (receptor) and is often based on docking scores or binding energies. While molecular docking can be performed independently, it was frequently used in conjunction with network pharmacology to validate compound–target predictions. In [S1], 6 of 22 studies employed docking analyses, all using candidate compounds and targets identified from prior network pharmacology screening. For example, in study A13, 93 compounds from Simiao Yong’an decoction were docked against five predicted target proteins: IL-17A, C3, C5a, VEGFR2, and STAT3, using docking scores to assess the interaction strength. As a result, glycyrrhizic acid showed a stronger predicted binding to IL-17A (−9.2 kcal/mol) than harpagide (−6.8 kcal/mol), supporting its prioritization as a candidate Q-marker. Additionally, because each compound was docked with five receptors, researchers identified the compounds with the lowest average docking scores, along with reference considerations, to select six compounds as Q-markers (19). Similarly, study A14 compared the docking scores of 12 candidate compounds with those of a reference drug (20). In contrast, the docking outcomes were less discriminative in studies A03 and A15. In A03, all five docked compounds had docking scores ≤ − 6.0 kcal/mol and were retained (15). In A15, although one compound (saikosaponin B2) failed to dock with any of the four targets, it was included based on its experimental activity (20).

#### 3.1.3 *In vitro/in vivo* studies

*In vitro* and *in vivo* experiments play critical roles in Q-marker identification by providing direct evidence of compound activity in laboratory environments, including animal bodies, cells, tissues, or biochemical assays. These experimental validations can be integrated at different stages of a Q-marker discovery strategy, depending on specific research objectives.

In [S1], 6 of 22 studies began with laboratory experiments, which included *in vivo* metabolomics and Caco-2 cell monolayer transport assays. Such early-stage experiments provided insights into the bioavailability, absorption, and pharmacodynamic relevance of candidate compounds, thus helping to prioritize those that were not only detectable in the extract but also biologically active and therapeutically meaningful. Mid-strategy laboratory experiments were conducted in 12 of the 22 studies. At this stage, experiments were performed to confirm initial predictions, explore mechanisms of action, optimize compound selection, and refine candidates before further analysis. Late-stage *in vitro* or *in vivo* studies, conducted in 7 studies, served as final validation steps to assess efficacy, safety, and translational potential.

For example, study A08 identified nine Q-marker candidates from Shuangshen Pingfei using UHPLC-ESI-QTOF-MS/MS and network pharmacology. These compounds were tested *in vitro* using bleomycin-treated macrophages, where all nine compounds reduced the expression of the inflammatory markers CCL2 and CCR2. Although none were excluded, this step provided confirmatory evidence of their anti-inflammatory potential (21). In study A04, 20 potential compounds were identified from Qingzao Jiufei Decoction via UHPLC-ESI-QTOF-MS and network pharmacology, and *in vivo* pharmacokinetic experiments revealed that two compounds were undetectable, likely due to poor bioavailability. Therefore, only 18 compounds were retained for further analysis (22). An integrative example is shown in study A02, in which 11 compounds were combined into an active compounds combination (ACC) based on their concentrations in the extract. Both *in vitro* (STC-1 cells) and *in vivo* (vincristine-induced paralytic ileus in rats) experiments demonstrated that ACC and the original extract had comparable effects in regulating bowel motility, healing, and reducing intestinal bleeding and weight loss. These findings validated the selected Q-markers and demonstrated that ACC could replicate the therapeutic efficacy of the full herbal extract (13).

#### 3.1.4 Literature review

Six of the 22 studies in [S1] incorporated additional literature-based criteria to further refine the selection of Q-markers. These studies included theories on TCM, toxicity profiles, and pharmacokinetic characteristics. This approach ensures that the proposed Q-markers align not only with experimental data but also with clinical relevance and principles of traditional usage. Most studies referred to the “five principles” of Q-marker determination as a widely accepted framework for final selection. These principles include (1): Traceability: Q-markers should be inherent secondary metabolites present in both raw materials and end products, enabling traceability throughout the quality control process (2); Specificity: Q-markers should be characteristic of the medicinal material, ideally unique to its species or closely related taxa, enabling clear differentiation from other herbal materials (3); Effectiveness: Q-markers must have clearly defined chemical structures and known biological or therapeutic activity (4); Measurability: Q-markers must be detectable and quantifiable through established analytical methods (5); Compatibility: Q-markers should align with TCM theory, prioritizing components in “monarch” herbs and considering those in “minister,” “assistant,” and “guide” herbs (5). However, not all studies applied all five principles uniformly. For example, studies A08, B09, B10, and D02 considered only the “compatibility” principle in the final Q-marker selection (21, 23–25). In study A07 with XKST, after a process of composition analysis, network pharmacology, plasma metabolomics, and validation experiments, nine components (danshensu, salvianolic acid A, salvianolic acid B, salvianolic acid D, ononin, quinic acid, puerarin, daidzein, and biochanin A) were included in the list of candidate Q-markers. Upon applying the “five principles,” salvianolic acid D, ononin, quinic acid, and biochanin A were excluded due to their low concentrations, which rendered them non-quantifiable. Danshensu, salvianolic acid B, and salvianolic acid A belong to the king herb of XKST, while puerarin and daidzein belong to the minister herb of XKST. Therefore, only these compounds were selected as the final Q-markers for XKST (12).

### 3.2 Strategy 2: profile–effect correlation strategy (24)

**Figure 4:**
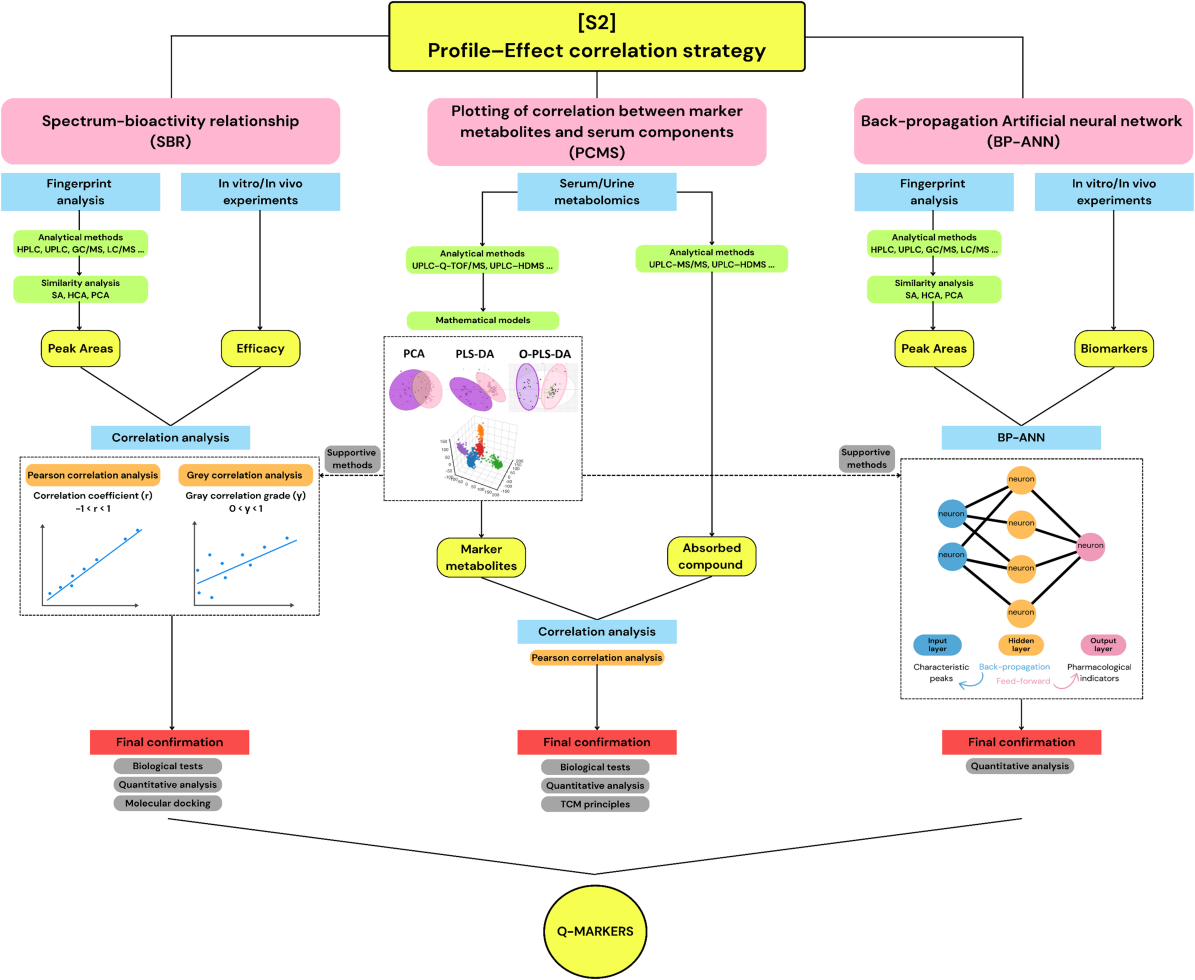
The demonstration of strategy 2: profile–effect correlation strategy.

Compared to [S1], the studies categorized under [S2] have also implemented fundamental steps, such as component analysis and laboratory experiments. However, network pharmacology was not utilized; instead, chemometric and mathematical analysis methods were emphasized for Q-marker identification. Upon detailed examination, the 24 studies in [S2] can be grouped into sub-strategies (1): the spectrum-bioactivity relationship approach (SBR) (8 cases, 33.4%) (2), the correlation between marker metabolites and serum components (PCMS) model (11 cases, 45.8%) (3), the artificial neural network (ANN) model (3 cases, 12.5%), and (4) others (2 cases, 8.3%) (Figure 4). The SBR group includes studies using chromatographic fingerprint peaks as the X variable and biological activity measurements as the Y variable, followed by statistical correlation analysis. Studies classified into the PCMS group use absorbed serum/urine components as the X variable and disease biomarkers or metabolomic changes as the Y variable, typically involving metabolomic profiling and correlation coefficient calculation. The ANN group includes studies using machine learning architecture with an input layer (chemical/chromatographic data), hidden layer (s), and output layer (biological responses), employing back-propagation or similar training algorithms. Although these sub-strategies differ in strategic detail, they require input from composition analyses and experimental data to evaluate the correlation between the extract components and therapeutic outcomes or metabolomic biomarkers. Based on these correlations, the compounds with the strongest associations were selected as Q-markers.

#### 3.2.1 Spectrum–bioactivity relationship strategy

This strategy integrates chromatographic fingerprinting with bioactivity evaluation to correlate specific chemical components of an herbal extract with its therapeutic effects. As shown in Table 3, all eight studies began with analytical techniques such as HPLC, UPLC, UHPLC, and two-dimensional liquid chromatography (2DLC) coupled with LTQ Orbitrap Mass Spectrometry (LTQ-Orbitrap-MS) to provide the chemical profile of the extracts, serving as the first input for SBR analysis. Subsequently, *in vitro* and *in vivo* assays were conducted to evaluate the biological activities of the extracts, including anti-inflammatory, antioxidant, and antimicrobial effects. The raw data from these experiments served as the second input for the SBR analysis. Statistical methods, such as Pearson correlation analysis or Grey correlation analysis (GCA), were then applied to identify the chromatographic peaks significantly associated with the observed bioactivities. Peaks with a high correlation coefficient (r) were considered potential Q-markers. Additionally, Partial Least Squares (PLS) models and their variants, including partial least squares regression (PLSR) or orthogonal partial least squares discriminant analysis (OPLS-DA), were sometimes used (5/8 cases) as supportive methods. These multivariate statistical techniques model the relationships between sets of variables. PLS constructs latent variables that maximize the covariance between predictor variables (X) and response variables (Y), enabling regression in PLSR and classification in OPLS-DA (26). Compounds with variable importance in projection (VIP) scores > 1 and *t*-tests indicating significant intergroup changes (*p* < 0.05) were identified as influential in the observed effects and, therefore, potential Q-markers. Finally, validation experiments such as *in vitro*/*in vivo* tests, quantitative analysis, or molecular docking were conducted (5/8 cases) to further confirm the Q-marker list.

**Table 3:** Strategy 2: profile–effect correlation strategy.

Code	Publication year	Materials	Target	Process					Q-marker results	Reference
				Step 1	Step 2	Step 3	Step 4	Step 5		
Sub-strategy: Spectrum-bioactivity relationship (SBR)										
B01	2020	Suhuang antitussive Capsule	Cough variant asthma	Fingerprint analysis: HPLC Similarity analysis: SA + HCA + PCA	Semi-quantitative analysis: HPLC-PDA	Stability studies: temperature, humidity	*In vitro*: RAW264.7 cells, anti-inflammatory activity		Praeruptorin A, schisandrin, arctiin and pseudoephedrine	(69)
B02	2020	Hugan Qingzhi Formula	Nonalcoholic fatty liver disease	UHPLC-QQQ-MS/MS	*In vitro*: LO2 cell model, lipid-lowering effect	SBR: Pearson correlation analysis	Validation: FFA-induced LO2 cells		Typhaneoside, isoquercitrin, and alisol B 23-acetate	(70)
B03	2021	Guizhi Fuling Prescription	Endometriosis	UPLC/Q-TOF-MS/MS	*In vivo*: Endometriosis rats	SBR: GCA	Molecular docking		Amygdalin, paeoniflorin, pentagalloyl glucose, cinnamic acid, and paeonol	(71)
B04	2022	Naoxintong Capsules	Thrombosis	Fingerprint analysis: UHPLC-PDA	*In vitro*: Thrombin/FXa inhibitory activity assay	SBR: Pearson correlation analysis + GCA + OPLS-DA	Validation: Thrombin/ FXa inhibitory and anticoagulant activities	Quantitative analysis: UHPLC–MS/MS-MRM-mode	Paeoniflorin, 3,5-dicaffeoylquinic acid, rosmarinic acid, lithospermic acid, salvianolic acid B, and Z-ligustilide	(72)
B05	2022	Bushen Huoxue Prescription	Diabetic retinopathy	Fingerprint analysis: HPLC-UV-ELSD Similarity analysis: SA + HCA	Component analysis: UPLC-Q-Exactive Orbitrap-MS	*In vivo*: HFD rats	SBR: PLSR + CCA	Validation: primary culture retinal Müller cells	Puerarin, daidzin, salvianolic acid B and ginsenoside Rb1	(73)
B06	2023	Kai-Xin-San	Amnesia	Fingerprint analysis: HPLC-UV-ELSD Similarity analysis: SA + HCA	Component analysis: UPLC-Q-Exactive Orbitrap-MS	*In vitro*: free radical scavenging activity	SBR: Pearson correlation analysis + GCA + OPLS-DA	Validation: SH-SY5Y cells, oxidative stress model	Sibiricose A6, ginsenosideRg1, and other 5 unknown compounds	(27)
B07	2023	Zishen Yutai Pill	Threatened abortion Quality control	Component analysis: 2DLC-LTQOrbitrap-MS	*In vitro*: HTR-8/SVneo cells, oxidative damage and migration models	*In vivo*: mouse endometrial receptivity disorder model and mouse premature ovarian failure model	SBR: PLS + PCA		4,5-Dicaffeoylquinic acid, hederagenin, malonyl-ginsenoside Rd., syringaresinol-O-β-D-glucopyranoside, 3-hydroxy-propionic acid tridecyl ester, dipsacus saponin R, 3,5-dicaffeoylquinic acid, foetidissimoside A isomer …	(74)
B08	2024	Nao An Capsules	Stroke	Fingerprint analysis: ALQFM + HPLC	Similarity analysis: HCA	*In vitro*: antioxidant activity	SBR: PLS + PCA	Literature review	Chlorogenic acid, hydroxysafflor yellow A, ferulic acid, ginsenoside Rc, ginsenoside Rb2	(75)
Sub-strategy: plotting of correlation between marker metabolites and serum components (PCMS)										
B09	2020	Sijunzi Decoction	Spleen qi deficiency syndrome	Biomarker collection: Serum/urine metabolomics + UPLC-Q-TOF/MS + PCA + OPLS-DA	Absorbed compound collection: Serum metabolomics + UPLC-MS/MS + PCA	PCMS: Pearson correlation analysis	TCM principle consideration: specificity, compatibility		Malonyl-ginsenoside Rb2, ginsenoside Ro, dehydrotumulosic acid, dihydroxy lanostene-triene-21-acid, glycyrrhizic acid, isoglabrolide, glycyrrhetnic acid, 2-atractylenolide	(23)
B10	2022	Wenxin Formula	Myocardial ischemia	Biomarker collection: Serum metabolomics + UPLC-HDMS + PCA + OPLS-DA	Absorbed compound collection: UPLC-G2-Si-MS/MS + Progenesis QI	PCMS: Pearson correlation analysis	TCM principle consideration: compatibility, traceability		Ginsenoside Rb1, cinnamic acid, paeoniflorin, and berberine	(24)
B11	2022	Wutou Decoction	Rheumatoid arthritis	Biomarker collection: Serum/urine metabolomics + UPLC-Q/TOF-MS + PCA + OPLS-DA	Absorbed compound collection: Serum metabolomics + UPLC-Q/TOF-MS + UNIFI	PCMS: Pearson correlation analysis			Aconitine, L-ephedrine, L-methylephedrine, quercetin, albiflorin, paeoniflorigenone, astragaline A, astragaloside II, glycyrrhetic acid, glycyrrhizic acid, licurazide, and isoliquiritigenin	(52)
B12	2022	Mailuoshutong pill	Thromboangiitis obliterans (Buerger’s disease)	Biomarker collection: Serum metabolomics + UHPLC-Q-Orbitrap HRMS + PCA + OPLS-DA	Absorbed compound collection: Serum metabolomics + UHPLC-Q-Orbitrap HRMS	PCMS: Pearson correlation analysis	Quantitative analysis: UPLC-MS/MS		Sweroside, chlorogenic acid, calycosin-7-glucoside, formononetin, paeoniflorin, liquiritigenin, and 3-butylidenephthalide	(76)
B13	2023	Baoyin Jian	Abnormal uterine bleeding	Biomarker collection: Urine metabolomics + PCA + OPLS-DA	Absorbed compound collection: Serum metabolomics + High-resolution UPLC-G2-Si/MSE	PCMS: Pearson correlation analysis	TCM principle consideration: traceability		Catalpol, rehmannioside D, phellodendrine, paeoniflorin, liquiritin, baicalin, berberine, asperosaponin VI, and glycyrrhetinic acid	(30)
B14	2023	Bushen Huoxue Prescription	Diabetic retinopathy	Biomarker collection: Urine metabolomics + UPLC-Q-Exactive Orbitrap MS + PCA + OPLS-DA	Absorbed compound collection: Serum metabolomics + UPLC-Q-Exactive Orbitrap MS	PCMS: Pearson correlation analysis	TCM principle consideration: specificity, traceability		Ajugol, protocatechuic acid, tanshinone IIA, panaxatriol and puerarin	(51)
B15	2023	Danggui Jianzhong Decoction	Primary dysmenorrhoea	Biomarker collection: Serum metabolomics + UPLC–HDMS + PCA + OPLS-DA	Absorbed compound collection: Serum metabolomics + UPLC–HDMS	PCMS: correlation analysis	TCM principle consideration: specificity		Ferulic acid, zizyphusin, and cinnamic acid	(77)
B16	2024	Danning Tablet	Cholestasis	Biomarker collection: Serum/liver metabolomics + LC/MS + PCA + OPLS-DA	Absorbed compound collection: Serum/liver metabolomics + LC/MS	PCMS: Pearson correlation analysis	Quantitative analysis: HPLC		Luteolin, kaempferol, apigenin, emodin, luteolin-7-glucoside, and 5,4′-dihydroxy-3,6,7,8,3′-pentamethoxyflavone	(78)
B17	2024	Danggui Buxue Decoction	Blood deficiency	Biomarker collection: Serum/urine metabolomics + UPLC-Q/TOF-MS + UPLC-MS + PCA + OPLS-DA	Absorbed compound collection: Serum metabolomics + UPLC-G2-Si/MSE	PCMS: Pearson correlation analysis	TCM principle consideration: stability, accessibility, and measurability		Calycosin-7-glucoside, ferulic acid, ligustilide, and astragaloside IV	(79)
B18	2024	Zhi-Zi-Hou-Po Decoction	Hepatorenal toxicity	Biomarker collection: plasma metabolomics + UHPLC-Q-Exactive Orbitrap-MS + PCA + OPLS-DA	Absorbed compound collection: Serum metabolomics + UHPLC-Q-Exactive Orbitrap-MS	PCMS: Pearson correlation analysis	Network pharmacology + Molecular docking	Validation: Real-time qPCR for marker expression	Naringin, hesperidin, neohesperidin, geniposide, genipin-1-β-D-gentiobioside, honokiol, magnolol, chlorogenic acid, and crocetin	(80)
B19	2024	Qifu Decoction	Heart failure	Biomarker collection: Serum, urine, and myocardial mitochondria metabolomics + UHPLC-Q-TOFMS + PCA + PLS-DA/OPLS-DA	Absorbed compound collection: Serum metabolomics + UHPLC-Q-TOFMS + SUS-plot	PCMS: Pearson correlation analysis	Validation: cell viability, western blot		Calycosin, neoline	(81)
Sub-strategy: Back-propagation Artificial neural network (BP-ANN)										
B20	2020	Herbal Pair	Migraine	Fingerprint analysis: HPLC-ESI-Q-TOF-MS/MS + SA + HCA + PCA	Biomarker selection: *in vivo*, nitroglycerin-induced migraine rats	BP-ANN + PLSR	Quantitative analysis: UPLC-MS/MS		Ferulic acid, senkyunolide A, 3-n-butylphthalide, Z-ligustilide, Z-3-butylidenephthalide, cyperotundone, nookatone, and α-cyperone	(34)
B21	2020	ShengMai Formula	Qi-Yin deficiency	Fingerprint analysis: HPLC + SA + HCA + PCA	Biomarker selection: *in vitro*, macrophage phagocytosis assay	BP-ANN + GRA + PLSR	Quantitative analysis: HPLC-UV		Schisandrol A, schisandrol B, methylophiopogonanone A, schisandrin B, ginsenoside Rf, ginsenoside Rb1, ginsenoside Rg2, and ginsenoside Rb2	(82)
B22	2021	Jinqi Jiangtang	Type 2 diabetes	Fingerprint analysis: UPLC-LTQ-Orbitrap + PCA	Biomarker selection: *in vitro*, glucose consumption, α-glucosidase inhibition, and glucose uptake assay	Chemical features ranking by ReliefF + BP-ANN			Berberine, palmatine, columbamine, jatrorrhizine, coptisine, epiberberine, berberubine, ononin, 1-O-caffeoylquinic acid, and demethyleneberberine	(35)
Others										
B23	2023	Yiqi Tongluo Capsule	Myocardial ischemia	UPLC-QTOF-MS	Serum metabolomics + UPLC-QTOF-MS	*In vitro*: H9c2 cell model	Literature review		Paeoniflorin, ferulic acid, calycosin, senkyunolide A, N-butylphthalide, Z-ligustilide, levistilide A, and astragaloside IV	(83)
B24	2024	Shengjiang Xiexin Decoction	Diarrhea	Plasma metabolomics + UHPLC-Q-Orbitrap HRMS	HCA, PCA	Molecular docking	*In vitro* binding affinity		Baicalin, baica lein, wogonoside, wogonin, liquiritigenin, isoliquiritigenin, norwogo nin, oroxylin A, dihydrobaicalin, chrysin, glycyrrhizic acid, glycyrrhetinic acid, oroxylin A 7-O-glucuronide, liquiritin, and iso liquiritin	(84)

For instance, in study B06, HPLC was used to generate chromatographic fingerprint profiles from 12 batches of Kai-Xin-San (KXS) extracts, identifying 25 common peaks with good consistency. These peaks were used as the first input variables for SBR. The antioxidant activity of each batch was evaluated using the DPPH free radical scavenging assay, and the resulting data served as the second input. Pearson’s correlation coefficients were calculated to determine the linear relationships between the peaks in the HPLC chromatograms and the antioxidant activities of the different batches. Thirteen peaks were positively correlated with DPPH free radical scavenging capacity (r > 0.7). In addition, OPLS-DA was applied using the same variables and revealed another 13 peaks with VIP values > 1. Finally, seven common peaks from both analyses were selected as Q-markers for KXS (27).

#### 3.2.2 Plotting of correlation between marker metabolites and serum components model

The PCMS model is a correlation-based analytical approach that evaluates the relationship between components absorbed into the bloodstream following administration and disease-related biomarkers. All 11 studies in this sub-strategy began with urine or serum metabolomic analyses to obtain profiles of the bioavailable herbal components and lists of potential biomarkers relevant to the disease under investigation. Here, principal component analysis (PCA) and the OPLS-DA mathematical model were used as pattern recognition tools. PCA reduces high-dimensional data into a smaller number of principal components, while OPLS-DA maximizes the separation between experimental groups by filtering out variability unrelated to the phenotype. This approach helps identify which absorbed components and biomarkers contribute most strongly to group discrimination (28). Subsequently, Pearson correlation analysis was used to calculate the correlation coefficient (r) between the serum components and marker metabolites, with higher correlation coefficients indicating stronger relationships (29). From this, lists of high-potential Q-markers and related biomarkers can be determined. For the validation step, 6/11 studies relied on literature reviews and TCM principles such as compatibility, specificity, and traceability to obtain the final Q-marker list, while 4/11 studies performed *in vitro* tests such as quantitative analysis, real-time qPCR, cell viability, and western blot.

In study B13, after establishing the abnormal uterine bleeding (AUB) model and treating with Baoyin Jian (BYJ), urine and serum samples were collected from control, model, and BYJ-treated rats. Urine metabolomic data were first analyzed using PCA, which revealed clear clustering within groups and notable separation between the control and model groups, indicating substantial metabolic alterations in the disease state. OPLS-DA was subsequently applied, and 32 ions with VIP scores > 1 and *p* < 0.05 (*t*-test) were identified as potential disease biomarkers. These metabolites served as the first variables in the PCMS model. In parallel, 59 serum components from BYJ-treated rats were characterized using the UPLC-G2-Si/MSE system combined with UNIFI software, representing the second variable. Pearson’s correlation analysis was used to calculate the correlation coefficients between the 59 serum compounds and the 32 biomarkers. Seven of the 59 compounds with _r_ > 0.7 and at least five biomarkers were ultimately identified as Q-markers of BYJ (30). In study B19, the Q-markers of Qifu decoction (QFD) for heart failure treatment were identified using a similar approach, with minor modifications in the biomarker selection strategy. A metabolomic analysis identified 21 serum biomarkers associated with heart failure, and only 14 that were significantly reversed by QFD treatment were selected as the initial input for PCMS. Simultaneously, 24 QFD-derived constituents were detected in the serum of the treated rats using UHPLC-Q-TOF-MS combined with shared and unique structures (SUS) plot analysis, which served as the second input. From Pearson correlation analysis, 11 compounds exhibiting strong correlations (|r| ≥ 0.6) were identified as potential bioactive components. Subsequent *in vitro* assays for cardioprotective activity confirmed that neoline and calycosin were potential Q-markers of QFD.

#### 3.2.3 Back-propagation artificial neural network model

ANNs are computational models inspired by the architecture of the human brain, which predict outcomes (outputs) based on input variables (predictors) using various training algorithms. In the reviewed studies, the back-propagation (BP) algorithm was the most commonly employed method for training ANN models (31). All cases within this sub-strategy specifically used the back-propagation artificial neural network (BP-ANN) approach.

A BP-ANN comprises an input layer, one or more hidden layers, and an output layer. Each layer consists of a set of interconnected nodes (neurons) linked by weights. The input layer receives raw data, which are then passed through the hidden layers. In these layers, each node calculates the weighted sum of its inputs and applies an activation function to introduce nonlinearity. The resulting information is then transmitted to the output layer. If the predicted output deviates from the actual result, the model enters the back-propagation phase, where the connection weights are iteratively adjusted to minimize the error between the predicted and observed outcomes. This training process continues until the network reaches an acceptable level of accuracy (32, 33). From the selected studies, ANN models required inputs of characteristic peak area data from herbal extracts (independent variables) and the corresponding bioactivity measurements (dependent variables). These data were used to compute specific indices, such as the degree of influence (ID) or mean impact values (MIVs), which contribute to the evaluation of the bioactive components. Finally, quantitative analyses such as UPLC-MS/MS or HPLC-UV were performed (in 2/3 cases) on the suggested compounds for reconfirmation.

For instance, in study B20, 18 chromatographic peaks from the herbal pair *Chuanxiong Rhizoma* and *Cyperi Rhizoma* extract (CRCR) were identified using HPLC-ESI-Q-TOF-MS/MS. Subsequently, the pharmacological effects of CRCR were evaluated in a migraine rat model by measuring six biomarkers: 5-hydroxytryptamine, calcitonin gene-related peptide, *β*-endorphin, vasoactive intestinal peptide, and nitric oxide synthase. A BP-ANN model was constructed with an input layer of 18 neurons (corresponding to the 18 characteristic peaks), a hidden layer of eight neurons, and an output layer comprising six neurons (representing the six pharmacological indicators). A sensitivity analysis was applied to compute the ID value, where variables with ID > 5% were considered to have significant effects on the pharmacological outcomes. Based on these criteria, 18 compounds were identified as candidate Q-markers. Additionally, a PLSR model was employed to further explore the correlation between chromatographic peaks and pharmacodynamic markers. This analysis identified 12 peaks with positive contributions across all six biomarkers. Finally, the eight compounds commonly identified by both the ANN and PLSR models were designated as Q-markers for CRCR (34). In study B22, a similar strategy was used to investigate the chemical profile and bioactivity of Jinqi Jiangtang. Instead of the PLSR model, a ReliefF-based feature selection algorithm was used to pre-select 18 representative compounds with potential antidiabetic activity. These compounds were then used as input variables in the ANN model, which consisted of 18 neurons in the input layer (peak intensities), 7 neurons in the hidden layer, and 3 neurons in the output layer (representing bioactivities). The MIV analysis revealed that 10 of the 18 preselected compounds with summed MIV > 0 were identified as potential Q-markers for Jinqi Jiangtang (35).

### 3.3 Strategy 3: *in silico* preliminary filtering strategy (8)

As shown in Table 4, the studies classified under [S3] adopted relatively simplified strategies, typically involving only two to three methodological steps. These approaches primarily relied on the *in vitro* determination of compound profiles, online databases, and computational tools for Q-marker identification without incorporating any validation experiments using cell lines or animal models. Analytical techniques, such as HPLC-MS or UPLC-MS, were commonly used to identify the chemical constituents (8 cases), which were subsequently analyzed through network pharmacology (7 cases), molecular docking (2 cases), or chemometric analyses, such as PCA and OPLS-DA (3 cases).

**Table 4:** Strategy 3: *in silico* preliminary filtering strategy.

Code	Publication year	Materials	Target	Process			Q-marker results	Reference
				Step 1	Step 2	Step 3		
C01	2021	Jie-Geng decoction	Airway inflammation and cough	Network pharmacology Compounds: TCMSP, BATMAN-TCM Compound targets: TCMSP, BATMAN-TCM, DrugBank, TTD, and CTD Disease targets: GeneCards, HPO, and CTD	HPLC-ELSD fingerprint	HCA and OPLS-DA	Glycyrrhizic acid, liquiritin, and platycodin D	(85)
C02	2021	Huo-Xue-Jiang-Tang Yin	Type 2 diabetes mellitus	HPLC-MS	Network pharmacology: Compound targets: TCMIP and SwissTargetPrediction Disease targets: OMIM and GeneCard		Gallic acid, rhmannioside D, hydroxysafflor yellow A, calycosin-7-O-β-D-glucoside, calycosin, astragaloside IV, astragaloside III, ophiopojaponin C, astragaloside II, isoastragaloside II, astragaloside I, and isoastragaloside I	(16)
C03	2021	Tangshen Formula	Diabetic nephropathy	UHPLC-Q-Orbitrap HRMS	Network pharmacology Compound targets: TCMSP, symmap databases, PubMed, and literature Disease targets: DisGeNet, GeneCards		Naringin, daidzein, genistein, formononetin, chlorogenic acid, aloe-emodin, nobiletin, tangeritin, ginsenoside Rg1, hesperetin, hesperidin, rhein, and limonin	(17)
C04	2022	San-Jiu-Wei-Tai Granules	Chronic gastritis	UPLC-QE-Orbitrap-MS	Network pharmacology: Compound target: SwissTargetPrediction Disease targets: OMIM, GeneCards	Molecular docking	2,6-Bis(4-ethylphenyl)perhydro-1,3,5,7-tetraoxanaphth-4-ylethane-1,2-diol, murrangatin, Meranzin hydrate, paeoniflorin, and albiflorin	(86)
C05	2023	Danggui Shaoyao San	Primary dysmenorrhea	UPLC–Q-TOF–MS	Network pharmacology: Compound targets: TCMSP, SwissTargetPrediction Disease targets: OMIM, GeneCards, and TTD	Molecular docking	Polyporenic acid C, senkyunolide P, alisol B 23-acetate, naringenin, gallic acid, ferulic acid, and albiflorin	(18)
C06	2024	Dajianzhong Decoction	Postoperative ileus	UPLC-QExactive-Orbitrap-MS	Fingerprint analysis: HPLC-TSQ-MS, HCA, PCA, and OPLS-DA	Network pharmacology: -Compound targets: PubChem, TCMSP, and SwissTargetPrediction Disease targets: OMIM, GeneCards, DisGeNET, GenCLiP3, and TTD	6-Gingerol, hydroxy-α-sanshool, hydroxy-β-sanshool, gingerenone A, ginsenoside Rb1, ginsenoside Rb2, ginsenoside Rb3, ginsenoside Rc, ginsenoside Rd., ginsenoside Re, ginsenoside Rf, and ginsenoside Rg1	(87)
C07	2024	Banxia-Houpo Decoction	Globus hystericus	UHPLC-QTOF-MS	Network pharmacology Compound targets: SwissTargetPrediction, TCMSP Disease target: GeneCards	Topological analysis	Honokiol, magnolol, magnoflorine, 6-gingerol, rosmarinic acid, and adenosine	(88)
C08	2024	Qianggan Capsule	Quality control	UHPLC-Q-TOF-MS/MS	LC-sMRM + PCA + OPLS-DA		Stachyose, paeoniflorin, gallic acid, lithospermic acid B, salvianolic acid, chlorogenic acid, danshensu, and albiflorin	(89)

### 3.4 Strategy 4: multi-criteria decision framework strategy (6)

**Figure 5:**
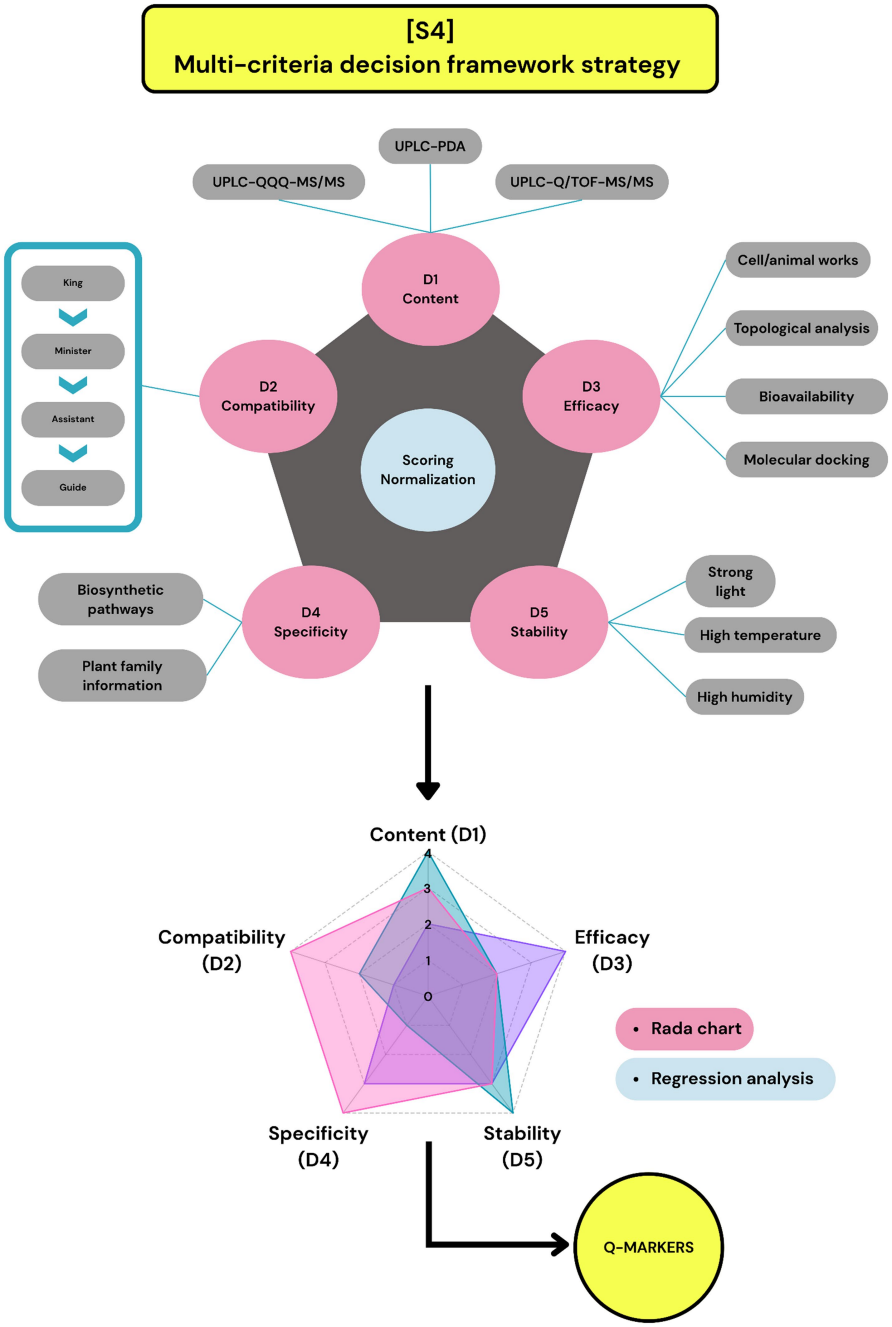
The demonstration of strategy 4: multi-criteria decision framework strategy.

This strategy follows the concept of the “five principles” of TCM (Traceability, Compatibility, Efficacy, Specificity, and Measurability) to determine Q-markers (5). This model implemented appropriate methodologies to generate datasets representing the essential properties of a Q-marker. In this model, each data set corresponded to one “variable” (dimension). After gathering the data for each dimension, the values were normalized to a “0–1 scale” or “1–4 scale” to mitigate the analytical variations caused by the disparity in the criteria of different dimensions. These normalized values were then integrated into a “multi-dimensional network,” in which the points corresponding to the different variables were interconnected. A regression analysis was then applied to compute a characteristic index for the network, such as the regression area (RA) or shaded area (QMI). Compounds with higher index values were considered to contribute more significantly to the therapeutic effects of the extract and were selected as Q-markers (Figure 5).

As shown in Table 5, the “content dimension” can be interpreted as representing both the measurability and traceability principles in Q-marker selection. For instance, in study D01, the content dimension was established directly through the quantitative analysis of compounds across multiple batches using UPLC-PDA (36). In contrast, study D03 integrated both metabolic and chemical profiling, selecting 19 compounds shown to enter the systemic circulation after administration, with their concentrations quantified in the prescription using UHPLC-Q/TOF-MS/MS analysis (37). Notably, although the “stability dimension” is not formally included in the “five principles” of TCM Q-marker selection, it was utilized in 4 out of 6 reviewed studies to construct multi-dimensional networks. This was based on the rationale that a stable Q-marker would strengthen the application of other principles and support the efficacy of the entire extract. In these studies, stability was assessed by measuring changes in the relative content of compounds under stress conditions, such as strong light, high temperature, and high humidity. However, inconsistencies have been observed in the interpretation of this attribute. While studies D01 and D04 prioritized highly stable compounds, D02 selected compounds exhibiting larger stability fluctuations, arguing that such instability may better reflect the intrinsic quality of a prescription (25, 36, 38). The “compatibility dimension” categorizes each compound according to its role in the TCM prescription hierarchy, namely, King, Minister, Assistant, or Guide herbs. King herbs target the primary disease and provide the main therapeutic action; minister herbs assist in enhancing or broadening this effect; assistant herbs help balance the formula or reduce side effects; and guide herbs direct the formula’s action to specific organs or channels, collectively contributing to a harmonized and effective formulation (39). The “efficacy dimension” evaluates the biological activity of candidate compounds, such as enzyme inhibition or molecular target binding. As shown in Table 5, five of six studies employed *in vitro* assays, while only study D05 utilized an *in vivo* model to assess the analgesic effects of the candidate compounds in mice (40). Furthermore, studies D02, D03, and D05 incorporated topological node degree values and molecular docking-derived binding affinities to enhance efficacy assessments (25, 37). Lastly, the “specificity dimension” was addressed only in study D05. To obtain such data, the biosynthetic pathways of the Q-marker candidates were analyzed, and information on the plant families to which each candidate compound belonged was collected. Compounds occurring in fewer plant families were considered more specific and were assigned correspondingly higher converted scores, in alignment with the principle that chemical specificity enhances the authenticity and traceability of herbal sources (40).

**Table 5:** Strategy 4: multi-criteria decision framework strategy.

Code	Publication year	Materials	Target	**Dimension**					Regression analysis	Q-markers	Reference
				Content dimension	Compatibility dimension	Efficacy dimension	Specificity dimension	Stability dimension			
D01	2020	Xuefu Zhuyu Capsule	Quality control	UPLC-PDA	N/A	*In vitro* antioxidant activity and anti-inflammatory activity	N/A	Change of relative contents under strong light, high temperature and high humidity	QMI: shaded area	Naringin, isoliquiritin, paeoniflorin, protocatechuic acid, neohesperidin, and ferulic acid	(36)
D02	2021	Shenqi Jiangtang Granule	Type 2 diabetes	UPLC-QQQ-MS/MS	TCM roles (King, Minister, Assistant, Guide)	*In vitro* inhibition rate, specific binding factor Degree value from topological analysis; Molecular docking	N/A	Change of relative contents under high temperature and high humidity	RA: regression area	Ginsenoside Re, ginsenoside Rd., ginsenoside Rg1, calycosin, ginsenoside Rb1, formononetin, astragaloside IV, ginsenoside Rf, ginsenoside Rc, notoginsenoside Fe, schisandrol A, and gomisin D	(25)
D03	2021	Qiliqiangxin Capsule	Chronic heart failure	UHPLC-Q/TOF-MS/MS	TCM roles (King, Minister, Assistant, Guide)	*In vitro* anti-heart failure effect Degree value from topological analysis; predicted *in vivo* bioavailability	N/A	N/A	CV: coefficient variation RA: regression area	Songorin, calycosin-7-O-β-D-glucopyranoside, astragaloside, tanshinone IIA, ginsenoside Re, hesperidin, and alisol A	(37)
D04	2022	Hedan Tablet	Hepatotoxicity	UPLC-PDA	N/A	*In vitro* antioxidant activity	N/A	Change of relative contents under strong light, high temperature and high humidity	QMI: shaded area	Salvianolic acid B, quercetin-3-O-glucuronide, isoquercitrin, hyperoside, psoralen, isopsoralen, psoralenoside, and isopsoralenoside	(38)
D05	2024	Tianshu Capsule	Migraine	HPLC-Q-TOF/MS	TCM roles (King, Minister, Assistant, Guide)	*In vivo* analgesic effect Degree value from topological analysis; Molecular docking	Specificity in plant families via biosynthetic pathways	Change of relative contents under high temperature, high humidity, and in work solutions	CV: coefficient variation RA: regression area	Gastrodin, senkyunolide I, senkyunolide A	(40)
D06	2024	Bu-Zhong-Yi-Qi-Tang	Quality control	UPLC-QqQ-MS/MS	N/A	*In vitro* immunomodulatory effect *In vivo* exposure (AUC0-t /dose) in plasma and in SIC	N/A	N/A	RA: regression area	Hesperidin, astragaloside IV, ononin, 18β-glycyrrhizic acid, narirutin, calycosin, cimigenoside, astragaloside II, and liquiritin	(90)

For instance, study D03 aimed to identify Q-markers in Qiliqiangxin capsules (QLQX) for the treatment of chronic heart failure. Based on a previous *in vivo* metabolomics investigation, 19 compounds were selected for their presence in the systemic circulation following administration. The “content dimension” was established by quantifying the concentrations of these 19 compounds in QLQX using UHPLC-Q/TOF-MS/MS, followed by normalization to a 4-point scale using a mathematical formula that accounted for the maximum and minimum values in the dataset. Sinapine thiocyanate, which had the highest average content (2363.13 μg/g), was assigned a score of 4, while ginsenoside Rb1 (286.75 μg/g) was normalized to 1.36. Subsequently, the “compatibility dimension” was quantified by assigning values according to TCM prescription hierarchy: compounds derived from king herbs were scored as 4, while those from minister, assistant, and guide herbs received scores of 3, 2, and 1, respectively. For example, formononetin from the king herb Astragali radix was assigned a value of 4, whereas alisol A from the assistant herb Alismatis rhizoma was assigned a value of 2. The “efficacy dimension” was evaluated via three factors. The anti-heart failure effects of the compounds were assessed using an isoprenaline-induced myocardial injury model in rat H9c2 cardiomyocytes, and their relative activity was normalized against the effective dose of QLQX. A topological analysis was also performed to calculate the DC of each compound in the compound–target interaction network of QLQX, along with their *in vivo*-predicted bioavailability retrieved through name-based queries in the TCMSP database. All dimensional values were normalized to a scale of 1–4, and the regression area (RA) and coefficient of variation (CV) were calculated for each compound. As a result, 7 compounds with RA ≥ 0.8× $\bar{\mathrm{RA}}$ and CV ≤ 1.2× $\bar{\mathrm{CV}}$ were selected as Q-markers of QLQX (37).

## 4 Discussion

Over the past 5 years, research on Q-markers in THPs has increased rapidly; however, methodological coherence remains lacking. Despite the growing number of studies, the field remains fragmented, with diverse strategies developed in isolation and lacking a unified conceptual framework to guide comparative evaluation (41). In comparison with previously published review articles on Q-marker identification, the present study offers several notable advances in scope and methodological rigor. First, whereas earlier works mainly adopt a narrative review format, our study represents the first systematic review of Q-marker research conducted according to PRISMA 2020 guidelines, allowing structured extraction, comparison, and evaluation of methodological details across studies. Second, by analyzing 60 original articles published between January 2020 and December 2024, this review provides an updated synthesis that captures new concepts and advanced analytical approaches that were not comprehensively discussed in previous reviews, including modern computational and statistical models such as similarity analysis (SA, HCA, PCA), correlation analysis (BCA, CCA, GRA, Pearson correlation), and advanced computational models like PLSR, OPLS-DA, and BP-ANN. Third, unlike prior reviews that focus narrowly on a single analytical domain (e.g., chromatographic analysis or network pharmacology), our work integrates all major analytical approaches currently used in Q-marker discovery and organizes them into a four-strategy classification system that reflects actual research practice. Finally, by critically examining the strengths and limitations of each strategy, this review proposes a refined and more operational framework for future Q-marker identification, offering clearer methodological guidance than has been available to date.

In this review, we classified 60 studies into four representative strategic models: [S1] mechanism-driven validation, [S2] profile–effect correlation modeling, [S3] *in silico* preliminary filtering, and [S4] multi-criteria decision frameworks. Strategies [S1], [S2], and [S3] typically follow a sequential logic, beginning with compound profiling, followed by computational or statistical filtering to narrow down candidate compounds. In contrast, [S4] adopts a concurrent evaluation model that integrates multiple criteria, such as bioavailability, efficacy, and prescription hierarchy, from the outset. Although common techniques such as network pharmacology, metabolomics, and experimental validation are used across all strategies, their specific roles and analytical weights differ substantially. More critically, no study has resolved the conflicting Q-marker results that emerge when different methods are applied. The absence of methodological interoperability is a central limitation of the current literature. To move forward, it is essential to reassess the internal logic of each strategy as well as its structural compatibility for future integrative models.

The mechanism-driven validation strategy ([S1]) exemplified the recurring structural inconsistencies observed in Q-marker research. This approach typically uses network pharmacology as an initial screening tool, followed by experimental assays to validate predicted compound–target interactions. Although several studies have reported biologically meaningful outcomes using this framework (42, 43), its overall methodological structure lacks consistency. Screening parameters, such as network centrality scores, oral bioavailability thresholds, and compound–target overlap ratios, are applied inconsistently across studies and are rarely grounded in statistically defined or empirically validated benchmarks (11, 44). Furthermore, most network pharmacology analyses rely on static network architectures derived from public databases, which cannot capture dynamic biological changes, such as time-dependent gene expression, post-translational modifications, or disease progression (45). Importantly, many databases are updated slowly and may not incorporate newly characterized compounds or targets, reducing their relevance for contemporary analyses. Moreover, newer databases or updated versions often build directly upon older resources, inheriting outdated or redundant information (46, 47). Because literature-derived entries frequently contain false positives or false negatives, researchers must critically evaluate data provenance and reliability when interpreting network pharmacology outputs. A further methodological concern, related to molecular docking, is the absence of a universal docking score cutoff to define “strong,” “medium,” or “weak” binding (15, 19). Studies apply widely varying thresholds, often based on convenience or precedent rather than validated correlations with experimental affinity. This inconsistency, combined with known variability between docking tools, limits the interpretability of docking results and highlights the need for experimental validation. The positioning of experimental validation varies; some studies incorporate it as an early filter for refining candidates, whereas others treat it as a downstream confirmation step detached from the screening logic. This asymmetry between prediction and validation undermines the internal coherence of [S1] and limits its reproducibility. To enhance the reliability of this strategy, future studies must establish clearer functional workflows and adopt standardized thresholds based on biological and pharmacological relevance.

The profile–effect correlation strategy ([S2]) offers a distinct contrast to the mechanism-focused strategy [S1] by emphasizing data-driven inference. It applies statistical and machine learning techniques to identify quantitative associations between chemical composition and pharmacological outcomes. Within this model, sub-strategies such as SBR, BP-ANN, and PCMS differ in their analytical structures. SBR typically uses PCA and PLS-DA to capture linear trends, whereas BP-ANN models nonlinear patterns through iterative training on labeled datasets (33, 48). Although a BP-ANN may yield a larger number of candidate markers, this does not guarantee higher accuracy, and the risks of overfitting and interpretability limitations remain significant (49, 50). Moreover, reliance on peak intensity data may bias the results toward high-abundance compounds, neglecting low-concentration constituents with pharmacological relevance (25). The PCMS addresses these issues by focusing on bioavailable serum compounds and correlating them with disease-related metabolic biomarkers to improve mechanistic plausibility. However, it may overlook active constituents below detection thresholds or those unrelated to the selected biomarkers. It is also worth noting the inconsistency in how different studies define correlation strength. Some studies categorized 0.6 < |r| < 0.7 as “high correlation” and |r| ≥ 0.7 as “extremely high correlation”; in other cases, 0.7 ≤ |r| < 0.8 indicated “high correlation” and 0.8 ≤ |r| ≤ 1 indicated “extremely high correlation” (51, 52). Thus, the predictive capacity of [S2] depends on model architecture as well as experimental design elements such as compound exposure, concentration bias, and biomarker specificity. To increase its reliability, [S2] may benefit from integrating multi-criteria frameworks, such as [S4], which account for both chemical relevance and biological context, thereby enabling a more balanced evaluation of Q-marker.

The *in silico* preliminary filtering strategy ([S3]) relied primarily on compound screening based on database mining, drug-likeness scoring, and molecular docking simulations. Although this model allows for the rapid triage of large chemical libraries with minimal experimental burden, its methodological simplicity often limits its strategic value. In contrast to [S1], which incorporates biological validation, and [S2], which relies on statistical correlation with experimental data, [S3] typically concludes at the computational prediction stage. Without empirical feedback, the functional relevance of predicted Q-marker candidates remains speculative. Furthermore, filtering thresholds such as docking scores or ADME criteria are often applied inconsistently across studies and are rarely justified by standardized benchmarks. Most critically, [S3] fails to address key pharmacological dimensions, such as systemic bioavailability, metabolic transformation, or multi-compound interactions inherent to traditional herbal formulations. These limitations hinder its use as an independent identification model. Instead, [S3] may be best positioned as an auxiliary pre-screening step within a broader analytical pipeline. Its operational speed can support early-stage filtering, but robust Q-marker evaluation ultimately requires integration with experimental validation, multi-criteria frameworks, and concepts more fully developed in strategy [S4].

The multi-criteria decision framework strategy ([S4]) adopts a structured scoring system to evaluate candidate Q-markers across multiple dimensions, including biological efficacy, content measurability, formulation role, chemical stability, and compound specificity. In contrast to single-criterion approaches, [S4] quantitatively integrates these variables, enabling a composite evaluation that reflects the overall functional relevance of each compound. Notably, the “efficacy” dimension incorporates experimental data from *in vitro* and *in vivo* studies, allowing for the inclusion of low-abundance but pharmacologically potent constituents (25, 53). In addition, [S4] is uniquely capable of capturing not only the direct effects of individual compounds but also their synergistic or supportive functions within the broader prescription matrix (54). For example, in the evaluation of Shenqi Jiangtang granules, astragaloside IV was selected as the final Q-marker because of its high efficacy score (28.31) despite a low content score (1.8), whereas ginsenosides Rh1 and Rb3, although abundant, were excluded due to insufficient bioactivity (25). Despite its strengths, [S4] faces several implementation challenges. The scoring criteria for each dimension are not standardized and often rely on literature-based or database-derived proxies instead of direct experimental validation. This leads to variability in scoring interpretations and weightings across studies (25, 36). The “compatibility” dimension, for instance, is frequently defined subjectively, while the “stability” dimension has been interpreted inconsistently; some studies prioritize stable compounds, whereas others emphasize instability as a proxy for formulation sensitivity. In fact, “stability” is a crucial dimension in Q-marker selection. According to the *WHO standards*, quality markers should remain chemically stable under defined storage conditions to ensure they function as reliable indicators (55). Unstable markers undermine batch-to-batch consistency, a core requirement for pharmaceutical quality control, and complicate manufacturing, storage, and shelf-life determination. Moreover, from a safety standpoint, chemically unstable constituents may degrade into reactive or even toxic metabolites under stress conditions. Additionally, [S4] demonstrates substantial value when used in combination with predictive models. In a study on Qiliqiangxin capsules, candidate compounds were initially screened using pharmacological criteria from [S2] and subsequently re-evaluated using [S4] scoring metrics, such as content, compatibility, and efficacy (37). This sequential integration allows researchers to rediscover low-abundance but functionally important compounds that would have been excluded by content-based screening alone.

Analysis of the average number of Q-markers identified across strategies revealed notable methodological differences. *In silico* preliminary filtering ([S3]) yielded the highest average number of Q-markers (8.25), likely reflecting its broad, hypothesis-generating nature and reliance on large-scale computational screening rather than empirical validation. In contrast, the profile–effect correlation strategy ([S2]) produced the lowest average number (6.61), consistent with its emphasis on statistically robust correlations that may filter out weaker or less consistent candidates. The mechanism-driven validation strategy [(S1)] showed a moderately high average (7.23), balancing biological plausibility with experimental confirmation. Similarly, the multi-criteria decision framework [(S4)] identified a comparable number of Q-markers (7.50) despite its more complex scoring and weighting system. Collectively, these patterns indicate that computationally oriented approaches tend to generate a broader set of putative markers, while experimentally grounded or statistically stringent methods yield more selective but potentially more reliable Q-marker sets.

**Figure 6:**
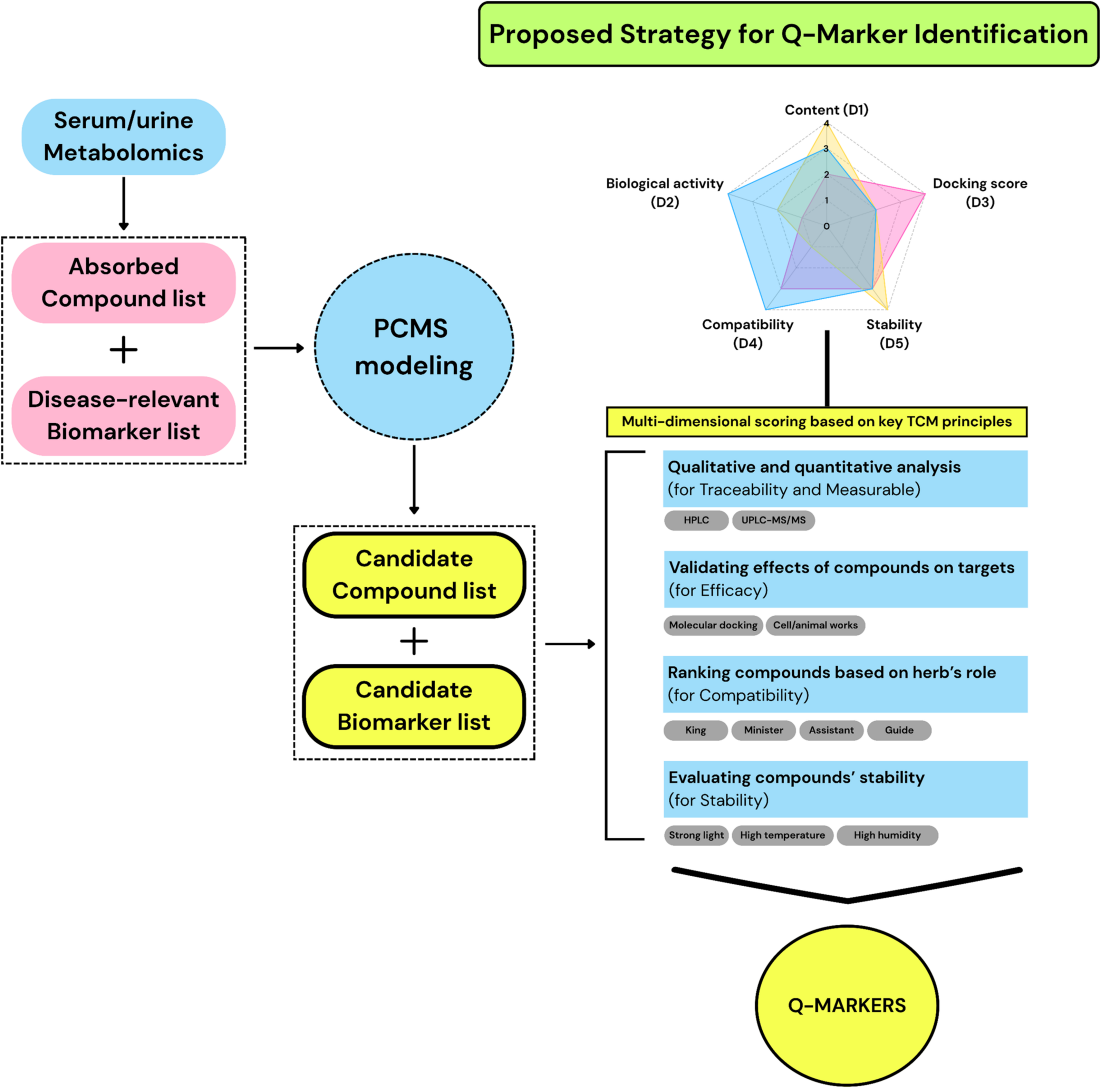
The demonstration of the proposed integrative strategy for Q-marker identification.

Based on the strengths and limitations identified across the current strategies (Table 6), we suggest an integrative strategy that future studies can benefit from through Q-marker identification to ensure both reliability and practicality (Figure 6). This strategy should begin with (1) the selection of candidate compounds detected in the serum or urine following *in vivo* administration to ensure bioavailability, followed by (2) the identification of disease-relevant biomarkers (3). PCMS modeling can then be used to evaluate compound–biomarker correlations, screening out important compounds and biomarkers (4). Finally, a multi-dimensional scoring system incorporating key TCM principles can be implemented: measurability (via analytical methods such as HPLC, UPLC-MS/MS), efficacy (via biological activity in *in vitro*/*in vivo* tests and molecular docking scores with candidate biomarkers), compatibility (via ranking of the original herb as King, Minister, Assistant, or Guide herb), and stability (via checking under strong light, high temperature, and high humidity). Study B10 can serve as a close example for the proposed strategy, which identified Q-markers of the Wenxin formula (WXF) for the treatment of cardiac disease (24). First, the authors focused on bioavailable components by identifying 37 WXF-derived compounds detected in serum after administration. Next, using UPLC-HDMS, 25 metabolites were identified as biomarkers for myocardial ischamia (MI). They then performed PCMS modeling with Pearson correlation analysis between serum constituents and MI-associated biomarkers, thereby identifying 8 active constituents highly related to the therapeutic effect. Finally, although not expressed as a formal scoring system, B10 implicitly evaluated these candidates according to some key TCM principles; for example, berberrubine-o-glucuronide was excluded because it is a compound generated by internal metabolism and is not an inherent component of the crude drugs. Ginsenoside Rb1, cinnamic acid, paeoniflorin, and berberine were selected as the Q-markers based on their traceability back to the crude drugs and their reflection of the compatibility principle of WXF’s formulation (representing the sovereign, minister, and assistant drugs). In fact, our proposed procedure is quite similar to that of the studies classified into the PCMS group of [S2] in this review, but in selecting the final Q-marker list based on TCM principles, we emphasize that the experimental methods should receive greater attention and be weighted equally using specific quantitative measures.

**Table 6:** Summary of the characteristics, advantages, and disadvantages of the four strategies.

Strategy	Frequency of use	Key characteristics	Advantages	Disadvantages	Average Q-marker number	Recommended use cases
[S1] Mechanism-driven validation strategy	22/60 cases (36.7%)	Uses network pharmacology to infer compound–target–disease links, followed by *in vitro/in vivo* validation	Supports biological mechanism interpretation Useful for multi-target analysis	Relies on potentially incomplete or outdated databases Assumptions and simplifications may not fully capture biological complexity May overlook dynamic biological changes	7.23	Mechanistic insight Pathway confirmation Traditional rationale Pilot screening
[S2] Profile–effect correlation strategy	24/60 cases (40.0%) (SBR: 33.4%; PCMS: 45.8%; ANN: 12.5%; others: 8.3%)	Uses statistical/machine learning models (e.g., SBR, PCMS, ANN) to correlate chemical profiles with pharmacological effects	Provides practical relevance High reliability	Requires complex statistical analysis and computational tools May overlook minor but active constituents	6.61	Functional screening Efficacy prediction Formulation optimization
[S3] *In silico* preliminary filtering strategy	8/60 cases (13.3%)	Conducts database mining, criteria filtering, and molecular docking without experimental validation	Rapid, simple Useful for initial triage	Lacks empirical validation Low reliability	8.25	Pilot screening Hypothesis generation Early-phase candidate narrowing
[S4] Multi-criteria decision framework strategy	6/60 cases (10.0%)	Integrates TCM’s principles (traceability, compatibility, efficacy, specificity, measurability) into a scoring model using indices such as RA or QMI	Provides holistic evaluation Aligns with TCM theory for evaluating Q-markers High reliability	Complexity in model weighting and normalization Inconsistent scoring criteria and lack of clear guidance	7.5	Quality control Regulatory benchmarking Comprehensive evaluation of complex formulas

This proposed strategy would address the major limitations of existing models by minimizing bias toward high-abundance compounds, ensuring biological relevance, and integrating data-driven predictions with experimental verification. As highlighted in the 2020 edition of the Chinese Pharmacopoeia and WHO standards, quality markers should reflect both chemical consistency and biological efficacy. Accordingly, the proposed framework aligns closely with international regulatory guidelines for herbal medicine quality control (55, 56). In particular, the bioavailability criterion ensures that selected Q-markers are not only present in raw materials but are also systemically absorbed, addressing the emphasis on transitivity and traceability throughout the production chain, from raw materials to finished products. Moreover, the multidimensional scoring system corresponds directly with the “five principles” framework advocated by Chinese regulatory authorities. From an industrial perspective, this framework offers systematic marker selection with regulatory-compliant documentation, provides robust evidence of biological activity, and facilitates traceability across all stages of production.

Notably, all 60 studies included in this review were conducted by research groups based in China, indicating that English-language databases effectively capture mainstream TCM research. However, as our search strategy was restricted to English-language articles, the potential consequences of excluding Chinese literature may include omitting specific methodological details available only in local databases. Therefore, future reviews should expand the scope to regional databases and additional languages to ensure a more comprehensive evaluation.

## 5 Conclusion

In conclusion, the 60 eligible studies published between 2020 and 2024 demonstrated increasing research activity, with the highest output in 2024. Four predominant methodological strategies were identified, with profile–effect correlation (S2) and mechanism-driven validation (S1) representing the majority of cases, followed by *in silico* preliminary filtering (S3) and multi-criteria decision frameworks (S4). The number of Q-markers identified varied across these strategies, ranging from an average of 6.61 Q-markers in S2 to 8.25 in S3, indicating clear quantitative differences in Q-marker outputs among the methodological approaches.

Although each strategy offers specific strengths, reliance on single, isolated methods often leads to inconsistent selection criteria and limited reproducibility. To advance Q-marker research, future studies should adopt more coherent, stepwise frameworks that integrate prediction, validation, and standardization. We therefore propose an integrated strategy combining (1) bioavailability-based compound selection (2), disease-relevant biomarker identification (3), correlation modeling, and (4) a multi-dimensional scoring system grounded in key TCM principles. This approach would provide a practical and reproducible foundation for improving Q-marker discovery and supporting quality control in traditional medicine.

## Data Availability

The raw data supporting the conclusions of this article will be made available by the authors, without undue reservation.

## Glossary

BP-ANN: Back-propagation artificial neural network
CV: Coefficient of variation
GC/MS: Gas chromatography–mass spectrometry
HCA: Hierarchical cluster analysis
HPLC: High-performance liquid chromatography
LC/MS: Liquid chromatography–mass spectrometry
LTQOrbitrap-MS: Linear trap quadrupole Orbitrap mass spectrometry
MIV: Mean impact value
MS/MS: Tandem mass spectrometry
OPLS-DA: Orthogonal partial least squares-discriminant analysis
PCA: Principal component analysis
PCMS: Plotting of correlation between marker metabolites and serum components
PLSR: partial least squares regression
Q/TOF: Quadrupole time-of-flight
Q-marker: Quality marker
QMI: Quality marker index
RA: Regression area
[S1]–[S4]: Strategy 1 to strategy 4
SBR: Spectrum–bioactivity relationship
TCM: Traditional Chinese medicine
TCMSP: Traditional Chinese Medicine Systems Pharmacology Database
THP: Traditional herbal prescription
UHPLC: Ultra-high-performance liquid chromatography
UHPLC-Q-Exactive-Orbitrap-MS: Ultra-high-performance liquid chromatography coupled with Q-Exactive Orbitrap mass spectrometry
UPLC: Ultra-performance liquid chromatography
VIP: Variable importance in projection
