# Supplementary material for: Q-marker identification strategies in traditional Chinese medicines: a systematic review of research from 2020 to 2024
Source: Front Med (Lausanne). 2026 Jan 16;12:1709969. doi: 10.3389/fmed.2025.1709969 (PMC12855425; doi:10.3389/fmed.2025.1709969)
Supplement: Supplementary file 1 [file Data_Sheet_1.PDF]

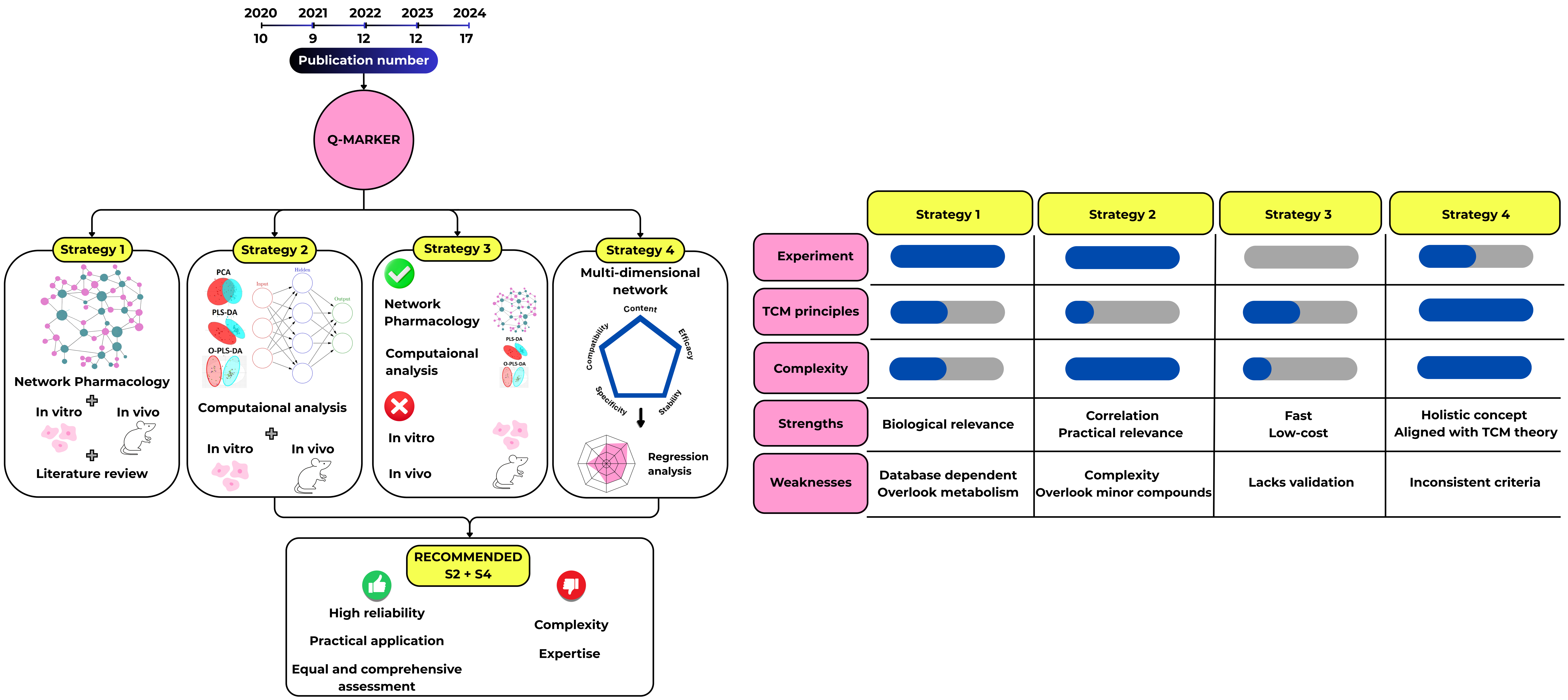
2020
2021
2022
2023
2024
10
9
12
12
17
Publication number
Q-MARKER
Strategy 1
Network Pharmacology
In vitro
In vivo
Literature review
Strategy 2
PCA
PLS-DA
O-PLS-DA
Input
Hidden
Output
Computaional analysis
In vitro
In vivo
Strategy 3
Network Pharmacology
Computaional analysis
PLS-DA
O-PLS-DA
In vitro
In vivo
Strategy 4
Multi-dimensional network
Content
Efficacy
Stability
Specificity
Compatibility
Regression analysis
RECOMMENDED S2 + S4
High reliability
Practical application
Equal and comprehensive assessment
Complexity
Expertise
Strategy 1
Strategy 2
Strategy 3
Strategy 4
Experiment
TCM principles
Complexity
Strengths
Biological relevance
Correlation
Practical relevance
Fast
Low-cost
Holistic concept
Aligned with TCM theory
Weaknesses
Database dependent
Overlook metabolism
Complexity
Overlook minor compounds
Lacks validation
Inconsistent criteria
