# Supplementary material for: Q-marker identification strategies in traditional Chinese medicines: a systematic review of research from 2020 to 2024
Source: Front Med (Lausanne). 2026 Jan 16;12:1709969. doi: 10.3389/fmed.2025.1709969 (PMC12855425; doi:10.3389/fmed.2025.1709969)
Supplement: Supplementary file 2 [file Data_Sheet_2.PDF]

Supplementary Materials

1. According to He et al. (2021), the regression area (RA) and coefficient variation (CV) are conculcated based on the Eqs. (2)–(4) formula (1):

$$D_i = \frac{3V_i}{V_{i\_max} - V_{i\_min}} \frac{V_{i\_max} - 4V_{i\_min}}{V_{i\_max} - V_{i\_min}} \tag{1}$$

$$RA = \frac{1}{2} * Sin\left(\frac{360}{n}\right)^{\circ} * \left[\sum_{i=1}^{n=1} \left(D_i D_{1+1} + D_n D_1\right)\right] (n \geq 3) \tag{2}$$

$$CV = \sqrt{\frac{\sum_{i=1}^{n} \left(D_i - \overline{D}\right)^2}{n-1}} / \overline{D} \tag{3}$$

$$\overline{D} = \frac{\sum_{i=1}^{n} D_i}{n} \tag{4}$$

Where

V: the variable corresponding to the characteristic data of the candidate Q-marker;

D: the standardized value of those variables;

RA: the regression area of the candidate Q-marker in the "radar chart" mode;

n: the number of dimensions in the radar chart;

CV: the coefficient of variation of the candidate Q-marker in the radar chart mode.

2. According to Wang et al. (2022), the index of Q-Marker (QMI) are conculcated based on the following formula (2):

$$p'_n = \sqrt{(CD_n)^2 + \left(R_{GSI(n)}\right)^2} + \sqrt{\left(R_{GSI(n)}\right)^2 + (AI_n)^2} + \sqrt{(CD_n)^2 + (AI_n)^2}$$

$$QMI_n = \sqrt{\frac{p'_n}{2}} \times \sqrt{\frac{p'_n}{2} - \sqrt{(CD_n)^2 + \left(R_{GSI(n)}\right)^2}} \times \sqrt{\frac{p'_n}{2} - \sqrt{\left(R_{GSI(n)}\right)^2 + (AI_n)^2}} \times \sqrt{\frac{p'_n}{2} - \sqrt{(CD_n)^2 + (AI_n)^2}}$$

Where

CD_n: the normalized value for constructing the content dimension of the tested compound n;

$R_{GSI(n)}$: the normalized value for constructing the stability dimension of the tested compound n;

AI_n: the activity index for the tested compound n;

P'_n: the perimeter of the triangle connected by CD_n, $R_{GSI(n)}$, and AI_n in the "spider-web" mode;

QMI_n: the shaded area of the triangle connected by CD_n, $R_{GSI(n)}$, and AI_n in the "spider-web" mode.

**These formula is applied to the case of a 3-dimensional radar chart and can be flexibly adjusted for different dimensions.*

References:

1. He L, Liu Y, Yang K, Zou Z, Fan C, Yao Z, et al. The Discovery of Q-Markers of Qiliqiangxin Capsule, a Traditional Chinese Medicine Prescription in the Treatment of Chronic Heart Failure, Based on a Novel Strategy of Multi-Dimensional "Radar Chart" Mode Evaluation. *Phytomedicine* (2021) 82:153443. Epub 20201223. doi: https://doi.org/10.1016/j.phymed.2020.153443.
2. Wang D, Ding J, Feng X, Chai X, Yang J, Liu C, et al. Identification of Q-Markers from Hedan Tablet by Employing "Spider-Web" Mode and Taking Compounds' Hepatotoxicity into Account. *Chin Herb Med* (2022) 14(4):612-21. doi: https://doi.org/10.1016/j.chmed.2021.08.007.
